# Bioactive Compounds in Infant Formula and Their Effects on Infant Nutrition and Health: A Systematic Literature Review

**DOI:** 10.1155/2021/8850080

**Published:** 2021-05-14

**Authors:** Cristine Couto Almeida, Bianca Figueiredo Mendonça Pereira, Katia Christina Leandro, Marion Pereira Costa, Bernardete Ferraz Spisso, Carlos Adam Conte-Junior

**Affiliations:** ^1^Instituto Nacional de Controle de Qualidade em Saúde, Fundação Oswaldo Cruz, Rio de Janeiro, Brazil; ^2^Centro Laboratorial Analítico, Faculdade de Medicina Veterinária, Universidade Federal Fluminense, Niterói, Brazil; ^3^Laboratório de Inspeção e Tecnologia de Leite e Derivados, Escola de Medicina Veterinária e Zootecnia, Universidade Federal da Bahia, Salvador, Brazil; ^4^Programa de Pós-Graduação em Ciência de Alimentos, Instituto de Química, Universidade Federal do Rio de Janeiro, Rio de Janeiro, Brazil

## Abstract

Infant formulas are an alternative to replace or supplement human milk when breastfeeding is not possible. The knowledge of human milk's bioactive compounds and their beneficial effects has attracted the interest of researchers in the field of infant nutrition, as well as researchers of technology and food sciences that seek to improve the nutritional characteristics of infant formulas. Several scientific studies evaluate the optimization of infant formula composition. The bioactive compound inclusion has been used to upgrade the quality and nutrition of infant formulas. In this context, the purpose of this systematic literature review is to assess the scientific evidence of bioactive compounds present in infant formulas (*α*-lactalbumin, lactoferrin, taurine, milk fat globule membrane, folates, polyamines, long-chain polyunsaturated fatty acids, prebiotics, and probiotics) and their effects on infant nutrition and health. Through previously determined criteria, studies published in the last fifteen years from five different databases were included to identify the advances in the optimization of infant formula composition. Over the last few years, there has been optimization of the infant formula composition, not only to increase the similarities in their content of macro and micronutrients but also to include novel bioactive ingredients with potential health benefits for infants. Although the infant food industry has advanced in the last years, there is no consensus on whether novel bioactive ingredients added to infant formulas have the same functional effects as the compounds found in human milk. Thus, further studies about the impact of bioactive compounds in infant nutrition are fundamental to infant health.

## 1. Introduction

Adequate nutrition during infancy and early childhood is essential to ensure children's optimal health, growth, and development [1, 2]. Malnutrition has been responsible, directly or indirectly, for 60% of annual deaths worldwide, often due to inappropriate feeding practices during the first year of life [3–5]. Human milk is universally recognized as the “gold standard” for feeding infants, providing readily bioavailable components and nutrients in a well-balanced supply, ensuring optimal growth and development for the child [6]. Breastfeeding offers innumerable benefits to both the mother and infant, and its short- and long-term consequences have already been scientifically proven. Among these benefits, we can mention the supply nutritional requirements and protection against diabetes [7, 8], the simple elimination of meconium [9], immunological components that prevent allergies [10, 11], decreased risk of jaundice [12], protection of the intestinal flora to avoid diarrhea, and protection against infections [13, 14]. Regarding the benefits for the mother, breastfeeding after childbirth makes the uterus return to normal size faster and decrease bleeding, preventing maternal anemia; accelerates weight loss; reduces the risk of breast, ovarian, and endometrial cancer; prevents osteoporosis; and protects against cardiovascular diseases, such as heart attack [15, 16].

The global public health recommends that infants be exclusively breastfed for the first six months of life to achieve optimal growth, development, and health. After this phase, they should receive complementary foods as they continue to be breastfed, at least until the age of two [6, 17–19]. However, in some situations, breastfeeding is not possible or advisable due to issues related to the mother's health [20–22] or due to the baby's health [23]. For these cases, international scientific medical societies recommend that when all strategies for maintaining breastfeeding are exhausted, the infant milk formulas should be used [20, 24]. Infant formulas used as a complement or a substitute for breast milk are the best alternative for child development compared to other unprocessed food sources because they can be manipulated to provide adequate nutrition. Infant formulas are products from cow's milk and other animals or vegetables or a mixture of these [25]. Cow's milk is the primary ingredient most often used in the manufacturing of these products. However, infant formula manufacturers seek to make cow's milk nutritional characteristics closer to human milk by chemically adjusting the macro and micronutrient composition [26–28]. In addition, human milk provides not only nutrient components but also potential bioactive compounds that perform many physiological functions other than nutrition, affecting the immune system, hormones and related compounds, antibacterial agents, enzymes, enzyme inhibitors, and encrypted peptides [29, 30]. Therefore, bioactive compounds are elements that “affect biological processes or substrates and hence have an impact on body function or condition and ultimately health” [31]. In human milk, these components come from various sources; some are produced and secreted in the mammary epithelium, while others are acquired due to maternal nutrition [29, 32–34]. There are many of these components in human milk; some are not yet identified; others, although already identified, do not yet have their physiological effects wholly understood. Therefore, their inclusion in infant formulas is not yet a technological reality.

In this way, the infant formula composition has been improved, not only to increase the nutritional similarities with human milk but also to include ingredients with additional benefits to infant health. These ingredients are bioactive compounds, which include proteins (*α*-lactalbumin and lactoferrin), milk fat globule membrane, taurine, folates (folic acid and 5-MTHF), polyamines, polyunsaturated fatty acids (docosahexanoic acid and arachidonic acid), prebiotics, and probiotics [35, 36]. The addition of these new ingredients has created novel scientific challenges not addressed by existing regulations. The current guidelines and legislations that evaluate the nutritional efficiency of ingredients added to infant formulas are not enough to guarantee the diversity of these novel compounds proposed by infant formula manufacturers [18]. To add any ingredients, it is necessary to follow the food safety standards that offer, as a basic premise, the “certain certainty of no harm” [37]. Thus, the addition of these bioactive compounds must be supported by a comprehensive assessment of their safety and efficacy since the “functional effect” is not always at all equivalent to a healthy effect [26, 38]. This systematic literature review is aimed at addressing the physiological health benefits of bioactive compounds currently incorporated in commercialized infant formulas and those already identified in human milk that have shown satisfactory results, demonstrating their potential for infant formula implementation. Besides, clinical studies highlighting the physiological effects of these bioactive compounds from human milk were addressed to demonstrate some beneficial effects.

## 2. Methodology

This systematic literature review recovered and assessed data available in four literature databases on the bioactive compounds identified in human milk and their effects on infant development and health and the potential to be implemented in infant formulas. To improve these systematic reviews, a four-phase flow diagram and the Preferred Reporting Items for Systematic Review (PRISMA) statement guidelines were used [39].

### 2.1. Systematic Search Methods

A literature search regardless of language was conducted on electronic databases using Medical Subject Headings (MeSH) terms: SciELO, ScienceDirect, PubMed/Medline, and Google Scholar. The screening process was performed in July 2020, with an interval filter set between 2005 and 2020, to identify the scientific advances related to the composition of infant formulas compared to human milk composition, focusing on bioactive compounds for the past fifteen years. For some specific topics related to clinical studies highlighting the beneficial effects of specific bioactive compounds, extending the publication date to include the original scientific data was necessary. Besides, we performed additional searching on the reference list of relevant articles/reviews identified through the initial screening. References considered essential to compose the revision were added, such as those that address the guidelines and legislation regarding infant formulas and global reports that portray the current scenario of infant nutrition.

The recovered papers of search sources were performed through a search string that summarizes the questions researched. The string was based on predetermined groups of keywords related to bioactive compounds and their potential benefits to infant nutrition and health, as shown in the search components (SC):

- *Search Components 1 (SC1)*. Bioactive compounds: “bioactive proteins” OR “caseins” OR “*α*-lactalbumin” OR “lactoferrin” OR “lysozyme” OR “secretory IgA” OR “taurine” OR “folates” OR “polyamines” OR “milk fat globule membrane” OR “docosahexaenoic acid” OR “arachidonic acid” OR “prebiotics” OR “probiotics”
- *Search Components 2 (SC2)*. Food matrix: “infant formula” OR “baby food” OR “human milk” OR “breast milk” OR “cow milk” OR “bovine milk”
- *Search Components 3 (SC3)*. Others: “*infant health*” OR “*infant nutrition*”

After retrieving the search component results, the Boolean operator “AND” was used to combine SC1, SC2, and SC3.

### 2.2. Research Question

The questions were formulated according to the population, intervention, comparison, and outcome (PICO) method. The following questions were formulated:

(P) Which bioactive compounds identified in human milk have been explored by the scientific community to be added to infant formulas so that they approximate the characteristics of human milk?

(I) What are the main physiological effects observed in bioactive compounds in infants?

(C) What are the similarities and differences between human milk and cow's milk used in the preparation of infant formulas concerning bioactive compounds (bioactive proteins, taurine, folates, polyamines, milk fat globule membrane, LC-PUFAs, prebiotic, and probiotic)?

(O) Clinical studies using formulas enriched with bioactive compounds (bioactive proteins, taurine, folates, polyamines, milk fat globule membrane, LC-PUFAs, prebiotic, and probiotic) demonstrate effects similar to those identified in exclusively breastfed infants?

### 2.3. Inclusion and Exclusion Criteria

Upon completing the literature search, the articles were sent to the MS Excel spreadsheet, which helped exclude replicas based on the title and for the organization of abstracts. Thus, a preliminary selection of scientific articles was carried out through abstracts, and some irrelevant articles were excluded. The eligible articles after the first screening were evaluated in more detail based on the description of their specific objectives, excluding those that are not relevant or that contained duplicate information or by the type of publication. The remaining papers' full texts were downloaded, read in full, and checked to examine the final inclusion criteria. Briefly, the eligibility assessment of references was carried out based on predefined inclusion and exclusion criteria:

- Were included original articles published in peer-reviewed journals that address bioactive compounds, infant formulas, child nutrition, and child health
- Were considered only studies dealing with bioactive compounds (bioactive proteins, milk fat globule membrane, taurine, folates, polyamines, polyunsaturated fatty acids, prebiotics, and probiotics) incorporated in infant formulas, as well as those compounds already identified in human milk that showed satisfactory results to be added to infant formulas
- Studies related to bioactive compounds identified in cow's milk, the primary raw material used in the preparation of infant formulas, and the human milk's bioactive characteristics were also considered in addressing the theme because they are relevant information in discussions
- Clinical studies highlighting the beneficial effects of these bioactive compounds in human milk on infant health and infant nutrition were included to justify their addition to infant formulas
- Editorials, letters, monographs, Master's dissertations, and Ph.D. theses were excluded
- Further, the articles with an unrelated topic, out of scope, duplicate content, missing data, or outside the eligibility criteria were excluded

### 2.4. Risk of Bias Assessment

Possible sources of bias include study inclusion/exclusion criteria and the impact of missing data, missing primary results, the chosen database, date, language, number of articles, and article type selected for this study.

### 2.5. Data Extraction

Two researchers (C.C.A and B.F.M.P.) extracted data from the included studies using a data extraction checklist that included the study's description (whether it was a theoretical study or a clinical study). Theoretical studies helped present and discuss the bioactive compounds present in human milk and cow's milk and the similarity between human milk and cow's milk used in elaborating infant formulas. Among these were selected review articles and book chapters. Clinical studies in animal or human models aided in the critical analysis of the inclusion of these bioactive compounds in infant formulas and their effects on infant development and health. Two tables were elaborated in which they report the bioactive functions related to the bioactive compounds described in this review and their respective authors responsible for the publication (Table S1) and a table describing the main relevant clinical findings related to the enrichment of infant formulas with bioactive compounds and their effects on infant health, comparing them to exclusively breastfed children and those who received standard formulas (without the addition of any bioactive compound) (Table S2). The clinical studies included in this systematic review were not accompanied by a meta-analysis since the heterogeneity of the groups was large, and it is not possible to compare them with each other.

### 2.6. Main Findings

**Figure 1:**
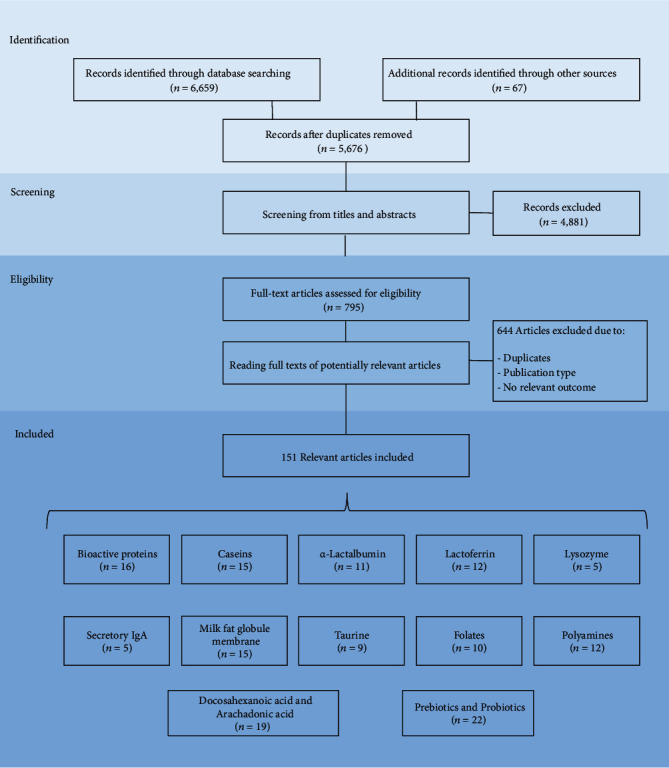
PRISMA flowchart of the processes followed in composing the systematic review.

The results of our systematic review were registered in a PRISMA flow diagram illustrated in Figure 1. This systematic search identified 52 papers at PubMed, 8 at Scielo, 1,017 at Science Direct, and 5,580 at Google Scholar. Besides, we manually added a further 67 articles updating the search on the databases totaling 6,726 papers. Of these, 1,050 were duplicates/triplicates and were excluded. A total of 5,676 remained after the exclusion of repeated articles. After reading the titles, abstracts, and full-text, only 151 papers were adequate for the current study purposes since they matched the eligibility criteria.

The articles found for bioactive protein, caseins, *α*-lactoalbumin, lactoferrin, lysozyme, secretory IgA, taurine, folates, polyamines, milk fat globule membrane, docosahexanoic acid, and arachadonic acid, as well as prebiotic and probiotic were analyzed separately. Thus, of the 151 research papers retrieved for qualitative synthesis, several investigated the bioactive proteins (*n* = 16), caseins (*n* = 15), *α*-lactoalbumin (*n* = 11), lactoferrin (*n* = 12), lysozyme (*n* = 5), secretory IgA (*n* = 5), taurine (*n* = 9), folates (*n* = 10), polyamines (*n* = 12), milk fat globule membrane (*n* = 15), docosahexanoic acid, and arachadonic acid (*n* = 19), as well as prebiotic and probiotic (*n* = 22).

## 3. Bioactive Compounds

Human milk contains a variety of nonnutritive compounds with specific bioactive characteristics that support different physiological functions in addition to simple nutrition. The knowledge of human milk's bioactive compounds and their beneficial effects has attracted the interest of researchers in the field of infant nutrition, as well as researchers of technology and food sciences that seek to improve the nutritional characteristics of human milk substitutes. Some of the bioactive compounds in human milk are already well identified and studied. However, although they have already been identified, thousands of others need to be clinically tested to prove their physiological effects on infant health and development.

The main commercial infant formulas are made with cow's milk. This raw material is easy to obtain and presents a low cost. Besides, cow's milk proved to be a valuable source of natural bioactive compounds, and clinical studies on infants that use formulations with cow's milk bioactive compounds have shown promising results. In addition to clinical studies, it is also important to evaluate these compounds' bioactivity and bioavailability in infant formula after undergoing thermal processing, such as the effects of packaging and storage.

A full description of all the bioactive compounds already identified or those that are supposedly present in human milk is beyond the scope of this review. In the following sections, we will address only the bioactive compounds currently incorporated in commercial infant formulas and others already identified that have shown satisfactory results, thus demonstrating the greater potential for implementation in infant formulas. Bioactive compounds, such as bioactive proteins, milk fat globule membrane, taurine, folates, polyamines, long-chain polyunsaturated fatty acids, prebiotic, and probiotics, can be incorporated into infant formulas to provide additional benefits to infant health. Some of these compounds added to infant formulas are controlled by regulatory agencies of the countries in which they are marketed (folic acid, taurine, LC-PUFAs, prebiotic, and probiotics, for example) for the safety and efficacy approval of each additive, while others (bioactive proteins, milk fat globule membrane, some folates, and polyamines) are still in the experimental stage of assessing their physiological effectiveness when added to infant formulas.

## 4. Bioactive Proteins

Bioactive proteins in human milk provide valuable biological functions in infants, and for this reason, they are the components with the greatest support from clinical trials to date [40, 41]. In addition to the biological functions derived from intact proteins and their amino acids, other functions emerge from the bioactive peptides formed during the digestion of caseins and whey proteins (*β*-casomorphins, *α*-lactorfin, *β*-lactorfin, albutensin A, *β*-lactotensin, lactoferricin, lactoferrampin, and others), as well as through the glycans that are released by glycoproteins, adding even more complexity to the functional properties of proteins [30, 34, 42, 43]. The evidence to date suggests that these peptides have opioid activities, antimicrobial, immunomodulatory, and other functions [43–45]. The release of these bioactive peptides begins inside the mammary gland by a complex array of proteases produced in the mother's milk [44, 46]. Moreover, these milk proteases continue to digest milk proteins within the infant's stomach. This reaction explains why breast milk proteins' digestion and absorption are effective in young infants with low protease activity [44, 47].

Cow's milk is a valuable source of natural bioactive compounds. For this reason, it has been widely studied as an alternative for inclusion into infant formulas [27]. They are not identical to their human counterparts; however, in many cases, the structures of bovine milk proteins share a high degree of homology with the human milk proteins, and because in vitro studies have shown equivalence between the bioactivities of human and bovine proteins, it is reasonable to study the effects of supplementation with these bovine proteins on infants [48, 49].

### 4.1. Caseins

Casein is present in human milk at low concentrations. Casein is not only a source of amino acids and trace elements (calcium, iron, and zinc) but also of bioactive peptides that break down and have an array of functions, including antimicrobial, gastrointestinal, immuno-modulating, and opioid activity [50–52]. Several peptides are formed during the proteolytic degradation of casein. The peptides derived from casein have numerous bioactive properties and are in an active state within the casein polypeptide chain. Human milk consists mainly of *β*- and *Κ*-caseins, with a lower concentration of *α*-casein [53].

Human k-casein (19 kDa) is a heavily glycosylated protein. It has been shown to prevent *Helicobacter pylori* from attaching to human gastric mucosa, which explains the lower incidence of *H. pylori* infections in breastfed babies [54]. The cleavage of k-casein results in the formation of a large carbohydrate-containing moiety, glycomacropeptide (GMP), which has been shown to have different biological effects, such as prebiotic effects and immunomodulatory activity, beyond the inhibition of pathogen adhesion to intestinal cells [55]. The evidence for these effects is more substantial in breastfed children [42]. In a study carried out by Bruck et al. [56], the authors showed that the bovine GMP present in the formula that was administered to rhesus monkeys infected with enteropathogenic *E. coli* had less severe diarrhea and of shorter duration when compared to those who received control formula, and also brought the intestinal microbiota closer to those of the monkeys breastfed with breast milk.

Beta-casein (27 kDa) is the major component of the human milk casein fraction. This protein can form various bioactive peptides (phosphopeptides or opioid-like compounds) before ingestion by infants or during digestion [50]. Casein phosphopeptides (CPPs) deriving from the digestion of milk proteins have been shown to chelate calcium and facilitate its intestinal absorption, as well as other minerals like iron and zinc [34, 48, 52]. Researchers have shown an increase in calcium absorption by bovine casein CPPs in animal experiments; however, absorption has not yet been observed in experiments with infant humans [48]. According to Lönnerdal [48], it is possible that the structures of human and bovine *β*-casein, which are similar but not identical, result in different affinities, which can affect absorption. In an in vitro study, Miquel et al. [57] investigated the formation and gastrointestinal survival of naturally occurring mineral carrier peptides released by simulated gastrointestinal digestion of infant formulas. The results suggest that the gastrointestinal digestion of infant formula promotes the formation of bioactive peptides with mineral carrier properties. However, this was not an in vivo study, so we cannot conclude that the same would occur in human infants or in models of animals. Several *β*-casein and *α*-casein peptide fragments have been shown to have opioid activities responsible for sleep and wake cycles, and they are necessary for the development and gastrointestinal function of infants [43]. Human milk contains biologically active opioid peptides derived from beta-casein, named *β*-casomorphins (BCMs). BCM peptides are released from human milk within the mammary gland before ingestion by the infant. It has been suggested that BCMs have an affinity to opioid receptors, conferring a wide array of physiological bioactivities, such as sleep induction, mucosal development, immunomodulatory, antioxidant, satiating, and gastrointestinal functions [50, 58]. Similar BCM peptides have been identified in *β*-casein from bovine milk [59]. According to Cattaneo et al. [60], some studies have reported the biological effects of bovine milk-derived BCM peptides in adults and infants. However, few effects were confirmed in model systems or animal trials. Limited information is available on the occurrence of BCMs in commercial infant formulas [61]. Jarmołowska and coworkers [62] assayed for opioid activity in samples of the formula for newborns available on the Polish market. The opioid activity of the peptides was determined by examining their influence on the motor activity of the isolated rabbit intestine. The results demonstrated that infant formulas containing predigested caseins as a protein source might be sources of BCMs. However, it is necessary to investigate the influence of an agonistic and antagonistic mixture of opioid peptides on the intestine's motor activity and evaluate the in vivo effects of these peptides on human physiology.

Alpha-casein is usually absent or present in low concentrations in human milk, unlike cow's milk, in which *α*-casein is the predominant casein [49]. The degradation of *α*-caseins can also generate CPPs. Studies have revealed critical biological activities of bovine *α*-casein. However, these biological activities are not observable in human milk *α*-casein, since it is present in such a low concentration [52].

### 4.2. Whey Proteins

Approximately 70% of human milk protein content consists of whey proteins. The main whey proteins, including *α*-lactalbumin (*α*-La), lactoferrin (Lf), lysozyme (Lz), secretory IgA (sIgA), and other minor protein, have been shown to have numerous bioactivities, as the growth and development of intestinal microflora, provide essential amino acids, and facilitated the digestion and the acquisition of nutrients from breast milk. It also plays an important role in immune function by providing a defense against pathogenic bacteria and viruses.

#### 4.2.1. *α*-Lactalbumin

Alpha-lactalbumin (14.2 kDa) is the most abundant protein, with 2–3 mg/mL concentration. The nutritional value of *α*-La lies in its high concentrations of essential amino acids, specifically tryptophan, cysteine, and lysine [40, 63, 64]. The presence of cysteine in this protein structure is related to strengthening the immune system, while the high levels of tryptophan help improve babies' sleep, mood, and cognitive development [43]. In addition, *α*-La has low allergenicity, and it is continuously used to enrich infant formulas [63, 65, 66]. During lactation, the mammary gland produces *α*-La and galactosyltransferase. These two proteins form the enzyme complex lactose synthase, which catalyzes lactose synthesis from glucose and galactose [63]. Besides, *α*-La has a specific binding site for calcium and another for essential trace elements, such as iron and zinc, which may facilitate its absorption [40, 67, 68]. Moreover, *α*-La becomes thermostable when bound to calcium and maybe glycosylated with mannose (Man), galactose (Gal), fucose (Fuc), glucose (Glc), and lactose (Lac). Compared to bovine *α*-La, approximately 10% is lactosylated, while the human milk protein does not change. Alpha-lactalbumin, when lactosylated, can prevent infection by inhibiting the binding of pathogens to the luminal surface of the intestinal epithelial cell due to the absence of lactosamine, which is necessary for its adhesion [56, 69, 70].

Alpha-lactalbumin accounts for 28% of the total protein in human milk and only 3% of the total protein in cow's milk [71]. Human and bovine *α*-La share a relatively similar percentage (73.9%) of homology in their amino acid sequences, and both consist of 123 amino acids. Advances in dairy milk separation technology have resulted in the development of a process that yields whey protein fractions with a substantially higher concentration of *α*-La than standard cow's whey and a reduction of *β*-La [63]. Enriched formulas with *α*-La can have lower protein levels than conventional formulas due to their higher protein quality since this whey fraction has an amino acid profile more similar to human milk [63, 67].

Fleddermann et al. [72] and Trabulsi et al. [71] evaluated the effect of a formula enriched with *α*-La on child growth of healthy term infants. Both studies revealed no difference in weight gain, weight for age, or weight for length compared to breastfed infants or infants fed with a standard formula. Another clinical study designed by Sandström et al. [68] compared the growth of breastfed infants and infants fed with *α*-La enriched formulas. The data demonstrated similar growth patterns among children. These clinical studies suggest that *α*-La, a source rich in essential amino acids, may play an ideal nutritional role. However, we cannot confirm that he can act as a programmer for the child's optimal growth.

Regarding the role played by *α*-La in the absorption of calcium and trace elements, although a clinical study carried out on young rhesus monkeys revealed that there was a good absorption of zinc and iron when administering infant formula supplemented with bovine *α*-lactalbumin, there are still no concrete studies that relate the effect of human *α*-La with the absorption of minerals in breastfed babies [73].

In addition to nutritional function, *α*-La is an important source of bioactive peptides, which may be related to gastrointestinal function. Evidence suggests that these benefits are likely to derive from bioactive peptides, from the content of tryptophan and cysteine, and potentially from the activity of posttranslational modifications, such as disulfide bridge or glycosylation [67]. Studies carried out on monkeys assessed the antimicrobial potential of *α*-La, revealing that its peptides have antimicrobial activity against Escherichia coli, Klebsiella pneumoniae, Staphylococcus aureus, Staphylococcus epidermidis, Streptococci, and Candida albicans. Since the primary structure of human *α*-La is similar to that of a monkey, it can be speculated that proteolysis of human *α*-La could produce the same antimicrobial peptides [40].

#### 4.2.2. Lactoferrin

Lactoferrin is an iron-binding glycoprotein of approximately 80 kDa containing 691 amino acids. Lactoferrin comprises 15%–20% total proteins; its concentration is higher in human colostrum (~7 mg/mL) and decreases during lactation [27]. It is synthesized by the mammary glands' epithelial cells, and it is also present in some exocrine fluids, such as saliva and tears. However, the highest levels are detected in milk secretions [74, 75]. Lactoferrin is a multifunctional protein and is considered an important host defense molecule involved in various biological functions [45, 76, 77].

Lactoferrin can perform all these functions because it contains a structure that partially resists proteolytic enzyme action, with a large part of lactoferrin found intact in breastfed children's intestines. Intact lactoferrin ensures better absorption of iron and other nutrients. Once the lactoferrin binds to specific receptors, it covers the surfaces of intestinal epithelial cells, stimulating and increasing the mucosal surface [10, 47, 78]. Lactoferrin can also exert bioactivities that are not mediated by binding to its receptor, such as bacteriostatic and bactericidal effects. Intact lactoferrin can exert bacteriostatic effects against *Escherichia coli*. This property results from the fact that lactoferrin has a high affinity for iron, allowing it to retain iron and prevent its availability to pathogens that require it, in addition to extracting it from bacteria, which ultimately prevents its use. Lactoferrin also has bactericidal activity against pathogens, such as *Vibrio cholera* and *Streptococcus mutans* [27, 79].

Lactoferrin was first identified in cow's milk and then isolated from human and bovine milk in several investigations [45, 80]. Human lactoferrin shares about 70% sequence homology with bovine lactoferrin, which has a molecular weight of 80 kDa and consists of 689 amino acids and antigenic determinants highly similar to its human counterpart; however, it was reported that infant formula enriched with lactoferrin does not improve iron absorption because bovine lactoferrin is not recognized by human lactoferrin receptors and is not present [81]. In addition, human milk contains a higher concentration of iron-bound whey protein than bovine milk, which can facilitate the absorption of iron from human milk. Despite this difference, both *in vitro* and animal models have demonstrated comparable bioactivity, including enhanced growth, near-identical functions against multiple pathogenic organisms, antibacterial and antiviral activity, antioxidant activity, and immunomodulation [74, 82, 83]. The similarities between human lactoferrin and bovine lactoferrin structures and functions indicate this fraction's potential utility in infant formula supplementation. The increasing commercial interest in exploiting lactoferrin's therapeutic value has stimulated the need for reliable concentration assays for its determination at endogenous levels in milk and colostrum, at supplemental levels in infant formulas, and at pharmaceutical levels in milk protein isolates [84]. Johnston et al. [74] evaluated the growth and tolerance of healthy infants who received formulas enriched with bovine lactoferrin in concentrations similar to that identified in mature human milk. The study revealed no difference in the growth rate with breastfed infants and that the formulations were well tolerated.

Bovine lactoferrin is commercially available, and it is relatively resistant to proteolysis. Infant formulas containing cow lactoferrin are currently under active study in a clinical trial (NCT#02103205) whose aim is to evaluate the effects of cow lactoferrin on the immune system, as well as the microbiota composition, metabolomics, growth, body composition, and cognitive development. In a study developed by Lönnerdal et al. [83], commercial cow lactoferrin added to infant formula was compared to human lactoferrin in an intestinal enterocyte model. The commercial cow lactoferrin was found to promote cell proliferation and differentiation similar to the lactoferrin present in human milk. However, the results showed no significant effect on the status of iron, infection, and the microflora of feces. It has recently been reported that many commercial sources of bovine lactoferrin contain significant amounts of lipopolysaccharides, which also have an affinity for lactoferrin receptors, blocking their bioactivities. When care was taken to use commercial bovine lactoferrin without contamination by lipopolysaccharides, in vitro studies yielded positive results on bovine lactoferrin's bioactivity (like the results for human lactoferrin) [85]. This shows that the bioactivities of contaminants can be prevented and that the purity of the bioactive compounds added to infant formula are relevant to the expected physiological effects. Manzoni et al. [86] compared infant formula with the addition of bovine lactoferrin (alone or in combination with probiotic *Lactobacillus rhamnosus* GG) with reduced late-onset sepsis in low birth weight newborns. The researchers demonstrated a significant reduction in the incidence of sepsis in premature infants who received formula supplemented with bovine lactoferrin and a lower prevalence of *Giardia* spp. and better growth compared to children who did not receive supplemented formulas. However, in untreated infants, the incidence rates of late-onset sepsis were similar between those fed exclusively with human milk and those fed exclusively with a standard formula. Given the high homology between human lactoferrin and bovine lactoferrin, it was argued that supplemented bovine lactoferrin overlaps human milk in its protection against sepsis. In a clinical study developed by King et al. [82], the authors assessed the impact of long-term feeding using formulas enriched with lactoferrin on the growth, hematology, immune parameters, and reduction in the incidence of respiratory diseases. The results showed a trend towards better weight gain up to 6 months of age and better hematological parameters. In addition, there were significantly fewer lower respiratory tract illnesses compared to infant fed regular formula.

The European Food Safety Authority recommends that 0–6-month-old infants take 1.2 g bovine lactoferrin daily without adverse effects. Changes in pH values and higher temperatures can modify lactoferrin's specific properties, such as the ability to bind to iron, which may explain the lower iron affinity of bovine lactoferrin compared to human lactoferrin, as reported by Aly et al. [81]. This is an issue that needs to be considered when assessing the presence of this compound in the finished product. In addition, due to the high cost of this ingredient and the difficulty in preserving the bioactive function of lactoferrin during infant formula production, the application of lactoferrin in commercial infant formulas is still limited [87].

#### 4.2.3. Lysozyme, Secretory IgA, and Other Minor Proteins

As well as lactoferrin, lysozyme also provides a strong defense to newborn due to their antimicrobial activity against a broad spectrum of bacteria, viruses, yeasts, fungi, and parasites, contributing to the development of an intestinal microbiota beneficial to infant health [88]. Lysozyme is an enzyme of about 14 kDa consisting of a polypeptide chain of 130 amino acids [89]. Lysozymes are widely distributed in bodily fluids, such as tears, saliva, blood, and other secretions and play an important role in the nonspecific defenses of an individual [89]. As well as lactoferrin, lysozyme is also found intact in the stool of breastfed infants in significant quantities and exert antimicrobial activity in the gut of breastfed infants [27, 48]. Lactoferrin and lysozyme can act synergistically to kill gram-negative bacteria that are normally resistant to bactericidal action [48]; however, lysozyme can also kill gram-positive bacteria by degrading the proteoglycan matrix of the bacterial cell wall [45]. It lyses mostly gram-positive and a few gram-negative bacteria, although gram-negative species appear to be more susceptible. Unlike other protective proteins in human milk, lysozyme concentrations steadily increase with prolonged lactation [89]. Compared to human milk, dairy animal milk has relatively lower levels of lysozyme. In human milk, the lysozyme concentration is about 370 and 240 *μ*g/mL in colostrum and mature milk, respectively, while in the milk of dairy animals, the concentration is practically undetectable (0.25 and 25 *μ*g/mL in bovine and goat milk, respectively) [32, 90].

Human milk contains five basic types of antibodies: IgA, IgM, IgD, and IgE. The major immunoglobulin fraction in human milk is IgA; however, in cow's milk, the IgG is present in a higher concentration than IgA, and the total immunoglobulin fraction is much lower than that in human milk [91, 92]. The IgA in human milk is presented in the form of two IgA molecules joined with a secretory component, being called secretory IgA (sIgA). sIgA is present in high concentrations during early lactation but remains in substantial concentrations throughout lactation [91]. This secretory component works as a defense mechanism for the antibody molecules, protecting them from gastric acid and digestive enzymes [89]. Maternal immunity can be transferred to the infant via antigen-specific sIgA in the mother's milk, thereby preventing adherence and penetration of both bacterial and dietary antigens capable of provoking inflammation in the intestinal mucosa [93]. Unlike most other antibodies, sIgA combats the disease without causing inflammation. Although the intestinal mucosa can produce sIgA to some extent in all infants, the amount of sIgA in breastfed infants far outweighs that of formula-fed infants [48]. Despite the differences in their structures, the IgG in cow's milk seems to have the same function observed in human milk [10, 78]. Some studies have been carried out to increase the concentration of immunoglobulins in infant formulas using immunoglobulins isolated from bovine colostrum. These studies' results were not satisfactory; moreover, designing this type of formula additive on a large scale is technologically questionable [92]. Although IgA antibodies in human milk cannot be reproduced in infant formula, other components, such as prebiotics, probiotics, and lactoferrin, can be added [35].

In addition to the proteins mentioned above, human milk contains many other minor bioactive proteins that exert diverse biological functions (Table 1). For example, the folate-binding protein can facilitate folate uptake; lipase and amylase are enzymes that can aid the digestion and utilization of some macro and micronutrients. Amylase can also aid the digestion of complex carbohydrates; *α*-antitrypsin and antichymotrypsin are protease inhibitors in human milk and work together to restrict the pancreatic proteases chymotrypsin and trypsin, in addition, to play a role in digestion and/or absorption of bioactive proteins present at relatively higher concentrations in colostrum; the growth factors, such as epidermal growth factor and insulin-like growth factors, originated from salivary glands of the infants or human milk are related to intestinal development; haptocorrin (vitamin B12 binding protein) is the main means for allowing vitamin B12 absorption in early infanthood; osteopontin affects intestinal and immune development, and some proteins that are inserted in milk fat globule membrane (mucin-1, butyrophilin, CD36, adipophilin, and lactadherin) that contribute to the antiviral and antibacterial activities of MGFM [29, 64, 89, 94].

## 5. Taurine

Taurine (2-aminoethane sulfonic acid) is a nonprotein amino acid found in most mammalian tissues, particularly in the brain, retina, myocardium, liver, skeletal muscle, kidney, and in human milk at all lactation stages (3.4–8.0 mg/100 mL) [18, 95]. Taurine is the product of the metabolism of methionine and cysteine and is not incorporated into any proteins. It is often referred to as a “nonessential” amino acid or a “conditionally essential.” This means that they can be considered essential in individual physiological states of development and certain clinical conditions [96]. In general, mammals can synthesize taurine endogenously, but some species, such as humans, are more dependent on taurine food sources [97]. Since humans have a relatively low ability to synthesize taurine, it is considered essential for normal perinatal development and is, therefore, nutritionally seen as a “conditionally essential” amino acid [98]. The biosynthesis of taurine varies according to the developmental stage. In this way, human infants, unlike adults, cannot synthesize taurine from methionine and cysteine precursors [95, 96, 99, 100]. Infants depend on taurine delivered from their mothers via either the placenta or human milk so that the plasma level remains significant [99]. Taurine is the most abundant free amino acid in human milk, representing approximately 50% of the total free amino acids, together with glutamic acid [101].

In an infant, taurine performs a wide variety of functions in the central nervous system, from development to neuroprotection. Also, it has other physiological functions, such as membrane stabilization, cell-volume regulation, mitochondrial protein translocation, antioxidative activity, and the modulation of intracellular calcium levels [98, 99]. Observational data suggest that relative taurine deficiency during the neonatal period is associated with adverse long-term neurodevelopmental outcomes in preterm and full-term infants. Based on this, Codex Alimentarius [102] recommends enriching all infant formula with taurine as a precautionary measure to provide improved nutrition with the same safety margin for their newly identified physiological functions as those found in human milk [103, 104].

The taurine contribution extends from conception and continues throughout life, but its most critical exposure period is during perinatal life. Perinatal taurine supplementation promotes prenatal and postnatal growth and development and protects against adult diseases, such as cardiomyopathy, renal dysfunction, developmental abnormalities, and severe damage to retinal neurons [98]. During pregnancy, taurine accumulates in the maternal tissues, where it is periodically released to the fetus via the placenta and to the newborn through breastfeeding [99]. It is accumulated mainly in the fetal and neonatal brain [95]. Even though it is well known that taurine is essential for fetuses and infants, the mechanism of action of taurine is not yet fully understood [95, 98, 99].

Some dairy products, such as cow's milk, have low amounts of taurine (0.5 mg/100 mL); for this reason, synthetic taurine has been added to infant formula [98]. In contrast to this, goat's milk, which has a similar taurine content to human milk, is a potential alternative to synthetic taurine [96]. Synthetic taurine is voluntarily added to infant formula as part of the wider strategy to match infant formula with human milk, as taurine is rare in cow's milk. Taurine concentrations in milk from mothers of term infants are around 4.7 mg/100 kcal [105]. The committee supports taurine's optional addition up to a maximum of 12 mg/100 mL to all types of formula without setting a minimum value [18]. In this way, taurine supplementation in infant formulas can improve infants' nourishment that is not breastfeeding [101]. Regarding its bioavailability in infant formulas, to date, no studies have evaluated the concentration of taurine after its production and storage.

In a systematic review performed by Verner et al. [103], the authors evaluated the growth and developmental effects of providing supplemental taurine to enterally or parenterally fed preterm or low birth weight infants. According to the authors' critical analysis, the available data from clinical trials revealed no evidence that supplementation with taurine or parenteral nutrition has significant clinical effects on premature or underweight infants' growth and development. The inherent limitation of these studies is that taurine's plasma levels after its supplementation in formulas were not conducted.

In a more updated systematic review and meta-analysis performed by Cao et al. [104], the authors evaluated different studies that analyzed the effect of taurine supplementation on growth in low birth weight infants. The evaluation of these studies concluded that there was no significant effect on growth in low birth weight infants. These data corroborate with the systematic review performed by Verner et al. [103]. Unlike the previous systematic review, the authors also presented the summary results of taurine on plasma. The meta-analysis results indicated that taurine supplementation significantly reduced length gain, plasma glycine, alanine, leucine, tyrosine, histidine, proline, and asparagine-glutamine. In addition, taurine supplementation has also been shown to affect the levels of acidic sterols, total fatty acids, total saturated fatty acids, and unsaturated fatty acids. However, according to the authors, although there are several significant differences in plasma indices, no significant effect on growth in low birth weight infants was observed with taurine supplementation.

Exogenously acquired taurine may not be essential to maintain levels in healthy children and may only be essential in very premature or seriously ill children. Despite the lack of evidence of these benefits in clinical trials, taurine is still added to infant formulas and parenteral nutrition solutions used to feed premature and low birth weight infants due to the association between taurine deficiency and its various adverse outcomes. It should be noted that most of the studies selected in both reviews were not recent. After the last publication period, we found no new studies that evaluate the benefits of taurine supplementation in infant formulas. Thus, further clinical studies are needed to evaluate the beneficial effects of taurine added to infant formulas.

In this way, considering the lack of scientific evidence for the benefit of the addition of taurine to infant formula, the panel of scientific opinion considers that there is no necessity to add taurine to infant formula. In addition, there are also no reports of adverse effects occurring with the current specifications of taurine in infant formula [6].

## 6. Folates

**Figure 2:**
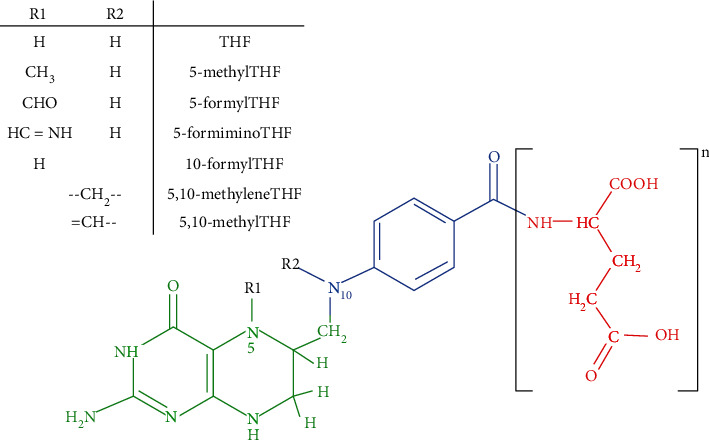
Folates are tripartite molecules, which consist of a pteridine ring (green), a para-aminobenzoate (blue), and a glutamate tail (red). A fully reduced tetrahydrofolate (THF) is present in these chemical structures. Figure adapted from Taylor and May [108].

Among the bioactive compounds related to vitamins, we focus on folates, a water-soluble B vitamin (B9) not synthesized in the human body (thus, its concentration in human milk depends on the mother's diet). In a regular diet, this nutrient comes mainly from foods of plant or microorganism origin. Folates are derived from tetrahydrofolate (THF), the most oxidized form consisting of a pteridine ring, a para-aminobenzoate, and a glutamate tail (Figure 2). Other folates differ in the length of their glutamate tails, ranging from one glutamate (monoglutamate) to approximately eight *γ*-linked L-glutamates (polyglutamate) and those with one-carbon attached to the molecule (methyl-, formyl-, methylene-, methenyl-, or formimino-) [106]. Folate is found in foods predominantly as polyglutamyl forms of tetrahydrofolate (THF), 5-methyl-THF, and 10-formyl-THF [107, 108].

In human milk, the average content of total folates is around 12.3 *μ*g/100 mL [106], while cow's milk contains low amounts (5–10 *μ*g/100 mL) and predominantly consists of the 5-methyl-THF form [107]. Folate is an essential vitamin that is involved in different biochemical processes. Its deficiency is a significant public health challenge since it can lead to physiological disorders, which can become aggravating in a developing child [109, 110]. Folate deficiency contributes to severe congenital anomalies, neural tube defects during embryogenesis, neurological diseases, and deficits in vitamin B12 that have negative consequences on the developing brain during infancy [111]. Folates are necessary for erythropoiesis, as they participate in the formation and maturation of red blood cells in the bone marrow. This is one of the causes associated with megaloblastic anemia and is usually due to insufficient intake of folates [112].

Folic acid (pteroyl-monoglutamic acid) is a synthetic and oxidized form of folate with a chemical structure and biological activities similar to those of other forms of folates. Synthetic folic acid is converted by dihydrofolate reductase enzyme into THF, the biochemically active form responsible for playing important cell replication and methylation reactions [113]. Folic acid is more stable and bioavailable than others; for this reason, it is commonly added into food supplements, such as infant formulas and fortified foods, to ensure adequate intake [114]. Considering the different bioavailability, dietary folate recommendations are expressed in units of dietary folate equivalents (DFE). Because the absorption efficiency of folates varies depending on their chemical form, DFE, this efficiency is defined as 1 DFE = 1 *μ*g food folates = 0.6 *μ*g folic acid from fortified food [113].

Folate intake recommendations for infants are based on the adequate folate intake estimated by the mean intake from exclusively breastfeed infants [113]. According to the European Food Safety Authority [6], a folate intake of 65 *μ*g DFE/day and 80 *μ*g DFE/day has proven to be adequate for the majority of infants in the first half and the second half of the first year of life, respectively. Folic acid is the only approved form for use in infant formulas. According to the European Commission Directive [18], all infant formulas must contain a minimum of 10 *μ*g/100 mL and a maximum of 50 *μ*g/100 mL folic acid [6, 37].

Although 5-methyl-THF is the predominant folate form in human milk and folinic acid, two other forms of folates have already been previously assessed to be used for fortification in specific food types; these folates are not currently approved for use in infant formulas [37, 115]. Troesch and coworkers [115] developed a recent study comparing infant formulas with the addition of 5-methyl-THF with a standard formula featuring folic acid fortification. This double-blind, randomized clinical study was carried out between two groups of infants. One group of infants received an infant formula containing 5-methyl-THF, and the others received infant formula containing folic acid. The effect on the growth, tolerability, and safety of the infants was evaluated. Infants who consumed the infant formula with 5-methyl-THF showed no significant differences in their growth and tolerance than the infants that consumed the formula with folic acid; its addition to the formulas also did not raise any safety concerns. Therefore, the authors concluded that there is no problem in allowing the addition of 5-methyl-THF as a source of folate in infant formulas (respecting the same concentrations allowed for folic acid).

When more exogenous folic acid is received than is required by the body, urinary excretion is increased, and the excess is eliminated. Toxicity is not a concern since folates are water-soluble and easily excreted by the kidneys when present in excess [116]. However, the consumption of high amounts of folic acid by subjects deficient in cobalamin (vitamin B12) increases the risk of neurological damage by masking the hematological manifestations of cobalamin deficiency, but this is not fully confirmed [117]. The bioaccessibility of folates must be considered since folates are thermolabile vitamins that can easily be lost during processing and storage. In a study developed by Yaman et al. [107], in which the bioaccessibility of folic acid added to infant formulas was evaluated through an in vitro digestibility analysis, the authors identified that the bioaccessibility of folic acid in infant formula decreases with a higher gastric pH. Thus, considering the different studies in vivo and *in vitro*, daily requirements should be reviewed to plan a novel formulation; besides being easily lost during processing, folic acid may also become less bioavailable due to the infant's gastric acid pH.

## 7. Polyamines

Human milk contains polyamines and biogenic amines, which belong to a group called bioactive molecules derived from amino acids. Polyamines are substances that play a significant role in regulating cell growth and proliferation, while biogenic amines are vasoactive or neuroactive [118]. Biologically active polyamines include putrescine (1,4-butane diamine), spermidine ((N-(3-aminopropyl)-1,4-butane diamine)), and spermine (N,N-bis (3-aminopropyl)-1,4-butane diamine). These molecules are considered as a separate group due to their biosynthetic pathways [119]. The polyamine oxidase (PAO) enzyme represents one of the key enzymes in polyamines' catabolic pathways. PAO catalyzes the oxidative deamination of spermidine or spermine, producing putrescine or spermidine, respectively. Diamine oxidase (DAO) catalyzes the biodegradation of putrescine, producing malondialdehyde (MDA). These enzymes involved in polyamine biosynthesis are exclusively located in the intracellular environment, where they participate in DNA transcription and RNA transduction [120]. Therefore, polyamines play an important role in cell proliferation, cell growth, protein synthesis, and nucleic acids [120, 121]. In addition to their endogenous synthesis, they are also supplied exogenously from dietary nutrients and can be found in animal foods, plant foods, and human milk in a free or conjugated form [122].

Human milk contains relatively high levels of polyamines, mainly spermine and spermidine, with a lower amount of putrescine; these are synthesized by lactating mammary epithelium [123–125], which is the first source of exogenous polyamines for a newborn [123]. The concentrations and profiles of these compounds depend on several factors, such as genetics, the stage of lactation in which the polyamines tend to decrease, the age of the mother, time of the day, the breast chose, the maternal polyamine dietary intake, and the mother's geographic location [122, 126, 127]. Besides, the concentration of polyamine in the human milk of preterm and term infants can also vary. In a comparative study, spermine, spermidine, and putrescine concentrations in human milk samples from mothers of preterm infants were 167.7 nmol/dL, 615.5 nmol/dL, and 165.6 nmol/dL, respectively. The counterparts in human milk samples from mothers of term infants were 173.4 nmol/dL, 457.5 nmol/dL, and 82.4 nmol/dL. Thus, in the human milk samples from mothers of term infants, putrescine was 50% lower, spermidine was 25% lower, and spermine remained practically unchanged. This result is consistent with the higher protein content in human milk from mothers of premature babies when we consider the role of polyamines in the stimulation of protein synthesis [123].

Bjelakovic et al. [120] investigated the polyamine metabolism in the colostrum (1st and 2nd day) and mature human milk (30th day of lactation) by measuring PAO and DAO enzyme activities, as well as by determining levels of MDA, the final product of polyamine biodegradation. The authors found a significant increase in PAO activity, which is responsible for the synthesis of spermidine and spermine, in the first days of lactation, and a marked decrease in DAO activity, thereby decreasing the concentration of putrescine and, consequently, the MDA levels throughout the first lactation month. This explains why the highest spermine and spermidine concentrations in human milk are detected in the first days of lactation. These data demonstrate the great importance of these bioactive compounds in the feeding of the newborn.

Although there are no recommendations for daily polyamine, even for adults, it is known that in stages of rapid cell growth, such as those that occur in the neonatal period, polyamine requirements are high [123, 124]. Thus, the ingestion of polyamines via human milk has an essential role in infant health. Polyamines are involved in the maturation of associated organs, such as the liver and pancreas, in the differentiation and the immune system's development. They can stimulate the proliferation and maturation of the gastrointestinal tract epithelium in newborns [120, 123, 125, 128]. In addition, polyamines can prevent food allergies in breastfed infants by decreasing mucosal permeability to antigenic proteins [129].

A recent review prepared by Muñoz-Esparza et al. [122], in which the authors reviewed the content of polyamines in food, exposed the contents of polyamines in human milk and cow's milk-based infant formulas reported in different studies. The content range of spermidine, spermine, and putrescine in human milk and infant formulas is shown in Table 2. According to the authors' analysis, the major polyamines in human milk are spermidine and spermine, and their contents differ significantly, with coefficients of variation of 68% and 53%, respectively. It is important to note that the human milk analyzed in these studies corresponds to different lactation phases, contributing to the high variability observed. In infant formula, the variability between different studies' results was even higher than that for human milk, with coefficients of variation of 89% for putrescine, 116% for spermidine, and 160% for spermine. Despite this variability, it can be concluded that the content and profiles of the polyamine in infant formula differ from those in human milk. In infant formula, the main polyamine is putrescine, whose content is higher than that of human milk, while its spermidine and spermine levels are lower. Studies related to the content of polyamine in human milk and infant formula are insufficient. More work is needed to clarify whether the variability observed in human milk and infant formula is due to different analytical methodologies or other factors that have not been sufficiently investigated.

Although the concentration of polyamines is higher in human milk than in cow's milk-based infant formulas, the bioactive function of these substances on the intestinal growth and epithelial permeability in neonates and infants remains an attractive hypothesis. However, this hypothesis remains highly controversial since polyamines present in cow's milk are not the same profile observed in human milk. The significant increase in the polyamine concentration in human milk during the first week of lactation also raises the question of polyamines' physiological impact on the neonate. The early polyamine concentrations likely reflect the enhanced metabolic activity and protein synthesis rate of the mammary glands [123, 127]. The use of infant formulas enriched with polyamines could have beneficial infant feeding applications during the first months of life, particularly in infant formulas for premature infants and infants who have early immune disorders [122].

The impact already reported on the bioactive effects of the polyamines present in human milk related to the maturation of the intestinal and systemic immune systems suggest that the supplementation of infant formulas manufactured with polyamines may improve the immunological functions of human infants in a similar way to that observed during breastfeeding [123, 126, 127]. Some studies have demonstrated that oral administration of polyamines induces early postnatal maturation of the intestines and acts on the repair of the intestinal mucosa and immune and inflammatory responses. However, most of these studies were developed with nonhuman animal models. According to Pérez-Cano et al. [130], spermine and spermidine administration improved the intestinal and systemic immune systems' maturation in suckling rats. In a study developed by Gómez-Gallego et al. [121], the authors evaluated the impact of infant formula supplementation with a mixture of different polyamines (putrescine, spermidine, and spermine) on the neonatal microbiota compositions in rats in the same concentrations present in human milk. The results demonstrated the potential effects of polyamines on the intestinal microbial composition of newborn rats. Another clinical study development by the same authors [131] investigated whether the proportion of polyamines found in human milk, administered in combination with commercial infant formula in early-weaned pups, affects the maturation of the immune system in a rat model. The results demonstrate that the supplementation of manufactured infant formula with polyamines enhanced the maturation of the systemic and intestinal immune system. These changes mainly relate to genes associated with immune system development. This study agreed with the results obtained by Pérez-Cano et al. [130]. The main difference between the former study compared to the latter is that the neonates were weaned early, and their only polyamine source was the enrichment of the infant formula. Despite rats and humans having some differences, the available data suggest similar immune development patterns [131].

We assume that similar processes may take place in infant humans. However, it is necessary to consider that if polyamines' profiles between different mothers and in the same mother are variable, the infant's microbiota composition and immune system development will also be different [127]. In this way, although the positive effects of polyamines on health were evidenced in rats, further clinical studies are necessary to verify if polyamines added to commercial infant formulas would have the same beneficial effects observed in the polyamines present in humans milk. Moreover, the doses and proportions of each of the polyamines that should be added to infant formula deserve a more thorough study. In addition to clinical studies, it is also necessary to consider infant formulas' processing and storage conditions, which can influence polyamines' content and profile [122, 131]. The lack of clinical studies explains why these bioactive compounds have not yet been incorporated into infant formulas.

## 8. Milk Fat Globule Membrane

The milk fat globule membrane (MFGM) is a complex structure composed mainly of lipids and proteins that surround the milk fat globule secreted by the alveolar epithelial cells of humans and other mammals. MFGM and its constituents are an important source of bioactive compounds. For this reason, in recent years, they have gained attention from researchers in the field of infant nutrition, who have shown great interest in their nutritional, physiological, and health benefits. Human and animal clinical studies have reported positive effects on immune and gastrointestinal health, brain development, and cognitive function. According to the studies, these effects were mainly attributed to the components of MFGM [132–134]. The lipid fraction of human milk constitutes 3 to 5% of its composition and is represented mainly by spherical globules, consisting of a “nucleus” of triglycerides (95-98% of total milk lipids) surrounded by a three-layer structural membrane composed of a complex mixture of polar lipids (phospholipids and sphingolipids) and nonpolar lipids (cholesterol and cerebrosides), specific proteins (mainly glycoproteins), and carbohydrates (Gangliosides) [135].

Regarding the lipids inserted in the MFGM, the polar lipids of the MFGM include phospholipids and sphingolipids. Phospholipids are complex mixtures of more than 30 molecular species of phosphatidylcholine, phosphatidylethanolamine, phosphatidylserine, phosphatidylinositol, and sphingophospholipids. However, 90% of all polar lipids are represented by phosphatidylcholine, phosphatidylethanolamine, and sphingophospholipids. Phosphatidylcholine acts as a precursor to the biosynthesis of membrane constituents, in addition to maintaining its permeability. Phosphatidylethanolamine is mainly linked to unsaturated fatty acids with high levels of oleic acid and linoleic acid [132, 136]. The sphingophospholipids are composed mostly of saturated fatty acids of medium or long-chain and have implications for many beneficial effects, such as neurodevelopment and intestinal development of newborns and protection against infections caused by bacteria [137, 138]. Among the apolar lipids, cholesterol works as a building block to MFGM, affecting myelin's development in the central and peripheral nervous systems. It helps as a substrate for the synthesis of bile acids, lipoproteins, vitamin D, hormones, and oxysterols that modulate cholesterol, lipids, and glucose homeostasis [139]. Human and bovine MFGM have similar sets of polar lipids but with different polar lipid molecules, in which the human MFGM contains more sphingomyelin than bovine; however, they have the same phospholipid composition [140]. Bovine MFGM contains considerable amounts of short-chain, saturated fatty acids (linoleic), and hardly any other long-chain polyunsaturated fatty acids (PUFA). However, human MFGM is rich in PUFAs, such as docosahexaenoic acid (DHA) [139, 141]. The carbohydrates present in MFGM are in the form of gangliosides, a group of glycosphingolipids comprised of sialic acid in their structure. They are known to be involved in neuronal growth, migration and maturation, neuritogenesis, synaptogenesis, and myelination [142].

The proteins contained in the fat globules are located in different layers within the membranes, with the glycoprotein carbohydrates directed outward (Hernell et al., [132]). Although quantitatively represent only 1% to 2% of the total protein content of human milk, MFGM contains more than four hundred minor proteins with various significant functions because many are known to have bioactive and potentially beneficial properties, especially about the defense mechanism of the child. Proteomic analysis of the MFGM identified 191 proteins, with functions enriched in metabolism/energy production (21%), cell communication (19%), and general transport (16%), and to a lesser degree immune response (20%) compared to whey proteins [64]. The main proteins identified so far as part of the human MFGM are xanthine oxidase, adipophilin, fatty acid-binding protein, and the heavily glycosylated proteins such as the mucins (mainly MUC-1 but also MUC-4, MUC-15, and others), lactadherin, CD36, and butyrophilin [141, 143]. Many of these proteins, such as mucins, lactaderine, and butyrophylline, in the glycosylated form. These glycoproteins play important roles in the intestinal microbiota defense mechanisms, acting as specific bacterial and viral receptors, protect infants from binding pathogens to glycan receptors on the mucosal cell surface, regulating and improving infant intestinal microbiota [104]. A comparative study between human MFGM and bovine MFGM revealed that bovine MFGM has similar amounts of proteins. The main bovine MFGM proteins include mucin 1, xanthine oxidase, CD36, butyrophylline, adipophylline, lactaderine, and fatty acid-binding protein [144, 145]. Moreover, it was identified that cow's milk whey proteins, especially beta-lactoglobulin, lactoferrin, and immunoglobulin, are commonly associated with MFGM [49, 140].

Maternal factors such as the lactation period, environmental conditions can influence the MFGM constituents [145–147]. Total milk fat increases with the stage of lactation and during breastfeeding. With the increase in total milk fat during the first month of lactation, there is an increase in the average size of MFGM diameter (0.2 to 15 *μ*m) and a reduction in phospholipids and cholesterol ratio to triacylglycerols. The larger the diameter of the globules, the larger the surface for efficient binding to lipolytic enzymes, thus facilitating the digestion and absorption of lipids in the infant gastrointestinal tract [141, 148]. Given this information, we could say that the human MFGM has better digestibility when compared to bovine MFGM. Concerning MFGM proteins, human colostrum has a higher concentration of glycosylated proteins (glycoproteins).

Standard formulas are devoid of MFGM during the production process, as the fat from cow's milk is replaced by vegetable oils [149]. Given the structure and composition of three unique layers, as well as the benefits observed in breastfed infants with those who receive standard formula and given the similarities between human and bovine MFGM and the bioactive properties of MFGM components, the supplementation of infant formulas with bovine MFGM would be a great alternative to narrow the gap between human breast milk and infant formulas made from cow's milk. Different clinical studies in animal and human models have shown positive results in child health and development by supplementing infant formulas with MFGM (mainly from cattle but also from other species), such as antiviral and antibacterial activities, anti-inflammatory activities, immune and gastrointestinal health, brain development, and cognitive function.

Timby and collaborators [150], in a randomized clinical trial developed with infants under two months of age, revealed that children who received experimental low-energy and low-protein infant formula supplemented with bovine MFGM showed an improvement in neurocognitive development and early growth when compared to those who received standard formula, and was not significantly different from those in the breastfed group. As part of the previous study, the same authors assessed whether enriching infant formulas with bovine MFGM would have a preventive effect on infections and their symptoms during the first year of life compared to breastfed babies [151]. The results revealed that supplementation with MFGM had an expected effect in decreasing the incidence of inflammatory diseases, similar to breastfed infants. These results confirm the role of MFGM proteins in defending against infections in breastfeeding infants.

There is ample evidence that MGFM components influence brain development, with huge differences compared to formula-fed infants [138, 152]. Polar lipid supplementation can reduce the gap in neuronal functions, such as cognitive performance, behavioral development, and myelination-promoting markers between breastfed and formula-fed infants. These findings support the idea that MFGM supplementation has a beneficial effect on neural functions throughout life. These neurodevelopment benefits may be associated with the MFGM ganglioside, considering the high ganglioside content in nervous tissue [153]. Gurnida et al. [152] also compared infants fed with an experimental formula enriched with gangliosides to a group fed standard formula. The authors observed an improvement in hand and eye coordination in those infants receiving the formula containing the gangliosides, and this improvement was correlated with an increase in serum ganglioside levels. In a recent large study, multiple neurodevelopmental tests were conducted to investigate infants' performance receiving infant formula enriched with MFGM-10 and lactoferrin. It was observed that infants receiving formula with added bovine MFGM and bovine lactoferrin had an accelerated neurodevelopmental profile at day 365 and improved language subcategories at day 545 [154].

Different studies have determined whether supplementation of MFGM in infant formula would lead to desirable metabolism and intestinal microbiota changes. Le Huërou-Luron et al. [155], in their study, identified that the incorporation of cow's milk fat and fragments of MFGM altered the developmental profile of the newborn intestine of pigs fed with formula. The addition of cow lipids also accelerated the maturation of the intestinal immune system, which was closer to that observed in mother-fed piglets [155]. According to the clinical study developed by Lee et al. [143], although the supplementation of MFGM suppressed microbial diversity and altered the metabolites associated with the microbiota, the authors did not observe significant changes in the composition of the fecal microbiota.

Some proteins present in the bovine MFGM have been shown to have broad activity against pathogens. Thus, a bovine whey protein concentrate enriched in the MFGM fraction may help prevent bacterial and viral diarrhea [156]. In their clinical study, Nelly et al. [156] demonstrated that the addition of a whey protein concentrate enriched with MFGM, supplied to infants, reduced the probability of an episode of bloody diarrhea and the prevalence of diarrhea.

The elaboration of a lipid complex with properties analogous to the human milk fat globule is an important way to reduce the gap between formula-fed and breastfed infants [132, 134]. The enrichment of infant formulas with bovine MFGM in different clinical studies increased the presence of phospholipids, sphingolipids, glycolipids, and glycoproteins with the benefits resulting from different results (especially immunological and cognitive results), with no reported adverse effects. Nevertheless, the precise mechanism of action of MFGM remains to be elucidated, as well as is necessary to discuss each component of MFGM to understand its physical, chemical, and nutritional characteristics [140]. Although there is a prototype of infant formula with bovine MFGM isolates, your inclusion in the manufacturing process of infant formulas is not yet carried out on a large scale, which is why it has not yet been adopted by the dairy industry [135]. Furthermore, there is no regulation for its addition to infant formulas.

## 9. Docosahexanoic Acid (DHA) and Arachidonic Acid (ARA)

The composition of essential fatty acids in infant feeding can also influence infants' metabolism and metabolic programming. Humans can synthesize saturated and monounsaturated fatty acids, but they cannot synthesize polyunsaturated fatty acids (PUFAs), such as *α*-linolenic acid (ALA-18: 3 *ω*-3) and linolenic acid (LA-18: 3 *ω*-6). These essential nutrients stand out for their body functions, but they must be present in the diet [157]. ALA and LA are precursors of long-chain polyunsaturated fatty acids (LC-PUFAs). ALA is converted to eicosapentaenoic acid (EPA, 20:5n-3) and then to docosahexaenoic acid (DHA, 22:6n-3), whereas LA is converted to arachidonic acid (ARA, 20:4n-6) [139, 157, 158]. This conversion of ALA into EPA and DHA depends on the individual metabolism of each mother. It is estimated that the conversion of ALA to EPA is around 0.2% to 6% and that approximately 63% of EPA is converted into DHA. Therefore, the formation of DHA is greater than that of EPA [159]. The reported mean DHA and ARA levels of human milk in mothers worldwide are 0.32% and 0.47% of total fatty acids, respectively [160]. DHA supplementation substantially increases human milk DHA content; in contrast, the response to ARA supplementation is more variable [161].

These two main LC-PUFAs (DHA and ARA) play a critical role in development and growth during pregnancy and early childhood. DHA and ARA constitute an integral structural part of the membranes of the cells of the central nervous system and retina [162, 163]. DHA is essential for the infant's brain normal growth and development, whereas DHA accumulates during the first years of life [164]. Like DHA, ARA is vital for infant neurological development, and, together, DHA and ARA account for approximately 25% of the fatty acids in the brain [165]. DHA is the main component of cell membranes and is the most abundant fatty acid in the brain and retina, making up about 40%–50% of PUFAs. ARA is a membrane component and a precursor to potent signaling molecules [166–169]. EPA, on the other hand, plays a more important role in cardiovascular and immunological health. These fatty acids accumulate in the retina, brain, and other neuronal tissues during pregnancy and in newborns and infants during the first two years of life, supplied through human milk [170]. This evidence demonstrates the importance of adequate intake of LC-PUFAs during pregnancy and lactation, which are critical neurological development windows [157, 171]. In early pregnancy, DHA and ARA are transferred from mother to fetus in the third trimester through the placenta [157]. Although human fetuses can synthesize DHA and ARA from their precursors, the amount of these fatty acids ranges widely between infants. In postnatal infants, human milk provides LC-PUFAs, such as DHA and ARA, and the precursors' LA and ALA, which are easily absorbed and readily used. However, the human milk of different mothers contains different amounts of these fatty acids. The ARA level is relatively consistent on a worldwide basis. Studies have concluded that most human milk ARA is derived from body stores, not from direct dietary absorption or as a result of synthesis from dietary LA [139].

In contrast, the level of DHA is more variable and depends on maternal diet and lifestyle. Thompkinson et al. [157] reported in their review that the synthesis of LC-PUFA from LA and ALA is more active at earlier gestational ages and decreases with advancing development. This leads us to believe that the concentration of LC-PUFA decreases during lactation. LC-PUFAs in human milk have received considerable attention because many of the bioactive effects in early life are mediated by these essential fatty acids. Since infants cannot synthesize LC-PUFA, the enrichment of infant formula with DHA and ARA is an effective way to promote the benefits of human milk in infants who cannot be breastfed, thereby guaranteeing adequate levels for normal development of the infant [158, 167, 172].

A blend of different fat sources can be present in infant formulas in such a way that the lipid composition closely resembles that of human milk. Presently, a mixture of vegetable oils, such as palm oil, coconut oil, sunflower oil, or soy oil, is added to infant formulas [158, 173, 174]. Different clinical studies have been carried out to evaluate the physiological effects of supplementing infant formulas with DHA and ARA and/or other types of lipids known to be present in human milk [161]. According to clinical studies developed by different authors, infants fed with infant formulas without the addition of LC-PUFAs had significantly lower levels of DHA and ARA in their plasma and red blood cells than those who were breastfed or fed with infant formula supplemented with DHA and ARA. In contrast, it was verified that the concentrations of DHA in the brains of infant breastfed are higher than those in the brains of infants fed formula [160]. A meta-analysis of randomized controlled trials was conducted to evaluate the effects of the supplementation of LC-PUFAs in infant formulas on infant development [175, 176]. Qawasmi et al. [176] examined LC-PUFA supplementation's efficacy in infant formulas on early cognitive functions, such as attention and memory. Simultaneously, Qawasmi et al. [175] assessed whether infant formula supplementation with LC-PUFAs could affect the infant's visual acuity. The meta-analysis demonstrated no significant effect of the supplementation of infant formula with LC-PUFA on infant cognition; in contrast, the evidence demonstrated a significant effect of LC-PUFA supplementation on the infants' visual acuity. According to Thompkinson and coworkers [172], the concentration of DHA is higher in plasma, erythrocyte membranes, and in the brains of infants that are breastfed or receive infant formulas supplemented with DHA compared to infants fed formula containing only the precursors' LA and ALA with no LC-PUFAs. According to a report by Rogers et al. [171], although synthetic capacities are functional in fetal and early neonatal life, most data indicate that the fetus or infant depends primarily on maternal sources or external supplies LC-PUFAs. Nonetheless, according to the available evidence, the results are inconclusive and need to be analyzed over the long term.

The inclusion of animal fats in infant formulas was widely used in the first part of the 20th century. However, these fats were replaced by vegetable oils, which provide higher levels of monounsaturated fatty acids and PUFAs and, consequently, yield better digestibility and be a cheaper and more easily accessible fat source [155, 177]. The interest in exploring the use of different fats from cow's milk for addition in infant formulas has increased in recent years, and relevant clinical studies have been developed [157]. Cow's milk fat contains a composition of fatty acids and lipid components different from those in vegetable oils [158]. Cow's milk fat can be added to infant formula in two different ways, either as anhydrous milk fat, containing triglycerides and other components like cholesterol and fat-soluble vitamins or as full-fat milk or cream, containing—besides triglycerides and cholesterol—all the components of the MFGM [158]. It is noteworthy that the cow's milk fat contains a low concentration of DHA and DHA compared to human milk and different sources of vegetable fat [155, 158]. In a study developed by Gianni et al. [178], in which the authors investigated the impact of a formula with a mixture of cow's milk fat and vegetable oils on the growth and gastrointestinal tolerance of healthy infants, there were no statistically significant differences in the weight, length, or head circumference growth rates among groups fed with a formula containing either a mix of dairy and vegetable oils or a blend of vegetable oils (with or without ARA and DHA). An infant formula that contains only vegetable oils contains lower levels of butyrate and medium-chain fatty acids and higher levels of monounsaturated fatty acids. For this reason, when a mixture of only vegetable fats is used, a source of palm oil needs to be added to reach a similar level of palmitic acid as that found in human milk [158].

According to the Codex Alimentarius and the Infant Formula Directive, the lipid concentration in standard infant formula must be no less than 3 g/100 kcal and not more than 6 g/100 kcal. Linoleic acid is allowed a range from 300 to 1,200 mg/100 kcal or 7%–20% of total fatty acids, which is similar to the amount found in average human milk. However, this concentration varies considerably with the mother's diet. The minimum level is well above what is required to prevent deficiency, and the maximum level considers that an overly high intake of LA may have adverse effects on several functions, such as lipoprotein metabolism, immune functions, eicosanoid balance, and lipid peroxidation. The addition of LC-PUFAs in infant formulas is optional. However, if these fatty acids are added to formulas, they are regulated by the maximum levels because overly high concentrations are not beneficial and may have harmful effects. The maximum level was set at 2% of total fatty acids for ARA and 1% for DHA. These maximum levels were set using the concentration in human milk as a reference. Furthermore, if added, there should be a proper balance between ARA and DHA, and the concentration of EPA should not exceed that of DHA, as EPA is a direct metabolic competitor of ARA, and DHA cannot exceed 0.5% of total fatty acids [18, 26, 179].

It is important to note that, regardless of the fat blend used, DHA and ARA are added as optional ingredients to infant formula [139, 161]. However, in infant formula marketed in European countries, the addition of DHA (20–50 mg/100 mL) is now mandatory [155]. According to Codex [102], the source of fat used to supplement infant formula must be considered because some countries expressly prohibit cotton and sesame seed oil and hydrogenated fats oils. Conditions for the use of fish oil in formula products have also been suggested to be an issue warranting discussion. Thus, given DHA and ARA's overall benefits, infant supplementation may improve neurological outcomes, especially in vulnerable populations. However, the optimal composition of the supplement and the dosing and treatment strategies still need to be determined to facilitate routine supplementation.

## 10. Prebiotic and Probiotic

### 10.1. Prebiotic

Prebiotics are defined as food ingredients that must reach the colon practically intact. These compounds should be fermented by a specific group of bacteria, stimulating its growth and/or activity and improving host health ([180, 181]). The most common prebiotics are nondigestible carbohydrates, ranging from disaccharides to polysaccharides. Human milk oligosaccharides (HMOs) are known to exert prebiotic effects that serve as a metabolic substrate for the desired bacteria and modulate an intestinal microbiota composition with health benefits for the breastfed infant [182–184]. HMOs are a family of structurally unconjugated glycans and quantitatively represent one of the main components of human milk. Many different functions have been identified for HMOs, such as their effects on microbiota composition by promoting desirable intestinal flora (since they are the substrates for beneficial bacteria in the gut, where they stimulate the growth of *Bifidobacterium* spp. and *Lactobacillus* spp.). HMOs also prevent pathogen adhesion since these compounds can act as receptor analogs of epithelial cells, acting as competitive ligands for pathogenic bacteria and their toxins, thereby preventing their adhesion; they also prevent infection and support immunity since a small amount can be absorbed into blood circulation. An example of this is their immunoreactivity modulation, thereby preventing allergic responses or food hypersensitivity [14, 185–187].

HMOs are soluble complexes composed of diverse saccharides comprised of five blocks: glucose (Glc), galactose (Gal), N-acetylglucosamine (GlcNAc), fucose (Fuc), or sialic acid (Neu5Ac) [182, 183, 188]. All HMOs are synthesized in the mammary glands, and their biosynthesis begins with the formation of a lactose core from galactose and glucose catalyzed by *β*-galactosyltransferase in the presence of *α*-lactalbumin. There is some variation among the HMOs from different mothers and the same mother, depending on the lactation stage. HMOs are present in higher concentrations during early lactation (20.9–23.0 mg/mL) than during late lactation (7.0–12.9 mg/mL) [183, 184]. The diversity among different mothers in their oligosaccharides' structures is also noteworthy since this diversity depends on the expression of specific transferase enzymes [189].

HMOs, exhibit a complex and dynamic mixture group, with sizes, structures, and functions different from each other. More than 200 different molecules have been identified and characterized in human milk, including many isomers. However, it is believed that human milk has more than a thousand different oligosaccharides, which represents a significant analytical challenge [118, 183, 190, 191]. According to Akkerman et al. [192], about 200 different types of HMOs have been characterized. HMOs' profiles and quantities depend on the mother's genetic characteristics, resulting in many types of oligosaccharides. The main oligosaccharides in human milk include fructo-oligosaccharides (FOS) and galacto-oligosaccharides (GOS), as well as glycololigosaccharides, isomalto-oligosaccharides, and xyloligosaccharides [13]. Oligosaccharides in the first few months of lactation contain about 10%–30% FOS and 70%–90% GOS [193]. FOS comprises a heterogeneous group of polymers composed of a linear chain formed by D-fructose molecules bound by glycosidic bonds and by D-glucose at one end. On the other hand, GOS are formed by D-galactose chains with a D-glucose residue at the reducing end [194]. GOS reaches the large intestine, where they can act as prebiotics [195]. For this reason, multiple studies focused on the effects of GOS in infant formula on microbiota composition, and the direct effects GOS can have on the epithelium or immune cells in the large intestine [194].

The HMOs are components of human milk and, therefore, are not found in the same composition and diversity in the milk of other animals [192]. Oligosaccharides have been detected in cow milk and some dairy products, but their physiological role is unclear [181]. According to Urashima et al. [196], 1 mg/mL of oligosaccharides was detected in the colostrum of cow's milk collected immediately after parturition. However, this concentration was reduced after 48 hours. Thus, oligosaccharides are practically nonexistent in the cow's milk used in the preparation of infant formulas. Consequently, cow's milk-based formulas are supplemented with nondigestible carbohydrates that have functional effects similar to those of some HMOs since synthetic HMOs' production is challenging and remains too expensive for broad application [190]. Currently, infant formulas derived from cow's milk are supplemented with nondigestible carbohydrates, such as galacto-oligosaccharides (GOS) and/or fructo-oligosaccharides (FOS) and/or polydextrose (PDX), which have been demonstrated to replace some of the functions of HMOs [197, 198]. Although these carbohydrate supplements have been reported to prevent allergies and atopic dermatitis, it is unclear how these effects are achieved [199, 200].

It is important to note that FOS and GOS are not like HMOs. These oligosaccharides of synthetic or vegetal origin are structurally different from HMOs, especially in monosaccharides' compositions. FOS and GOS polymers of galactose and fructose, respectively, do not contain fucose or N-acetylglycosamine like HMOs [192]. FOS can be produced enzymatically from inulin obtained from natural sources, such as chicory and sugar beet [201]. In contrast, commercial GOS are generally obtained by *β*-galactosidases of bacterial and fungal origin [202]. Studies have shown an improvement in intestinal flora composition, the frequency of depositions, and softer stool consistency by using formulas supplemented with long-chain fructan polysaccharides, such as inulin and FOS, and mixtures of GOS in healthy newborns [203].

The prebiotic effects of other milk bioactive glycans, such as glycoproteins and glycolipids, are also recognized as responsible for the development of the microbiota, with direct application in the prevention of diseases, such as necrotizing enterocolitis, a common and devastating disease of preterm infants [180]. Glycans linked to lactoferrin, as already mentioned, provide antibacterial and antiviral activities in the intestine through direct effects on pathogens, affecting gastrointestinal and immune functions [27]. Cow's milk lacks specific bioactive glycans that are presumably important for child development [77, 200, 204]. A more in-depth study on identifying bioactive glycans in bovine milk would be of great importance for manipulating infant formulas since human milk is an unviable source for commercialization. A likely source of such glycans would be from other species of animals [180].

Simple and complex oligosaccharides are present in cow's milk. Despite the structural similarity, the concentration is significantly lower than in mature human milk, decreasing throughout lactation [180]. Barile et al. [180] demonstrated a way to isolate oligosaccharides from bovine milk from dairy by-products using membrane filtration was demonstrated and revealed that seven of the fifteen identified oligosaccharides have the same composition as HMOs. Given the extremely large and growing production of cheese whey worldwide, its use can provide a significant extraction source for HMO imitators. This research line would add a new dimension to the profitable use of whey in the dairy industry.

### 10.2. Probiotic

When ingested at defined doses, probiotics are living bacteria that affect the host beneficially by improving his or her intestinal microbiological balance [205]. *Bifidobacterium* spp. and *Lactobacillus* spp. are the most common probiotic bacteria found in infants' guts [29]. Through a mechanism called competitive exclusion, probiotic microorganisms allow the intestinal microbiota to be modulated, preventing the colonization of the mucosa via potentially pathogenic microorganisms through competition for adhesion and nutrient sites and/or through the production of antimicrobial compounds [206, 207]. Probiotic microorganisms also seem to influence the bioavailability and digestibility of lipids and proteins due to the release of various enzymes in the intestinal lumen [208]. The commonly administered probiotic bacteria belong to the genera *Bifidobacterium* and *Lactobacillus* but can be provided either as single or as mixtures of strains [209].

Several clinical studies have compared the intestinal flora of breastfed infants with infants who received infant formulas. These studies have shown that breastfed infants' intestinal flora had higher proportions of *Bifidobacterium* and *Lactobacillus* than formula-fed infants. They presented a more complex flora with higher proportions of *Bacteroides*, *Enterobacteriaceae*, and *Clostridium* [192, 203, 210, 211]. Lactobacilli and bifidobacteria are the most common target genera for prebiotics; however, bifidobacteria changes are more likely to be compared to lactobacilli. This may be because more bifidobacteria usually reside in the human infant colon than lactobacilli, and they exhibit a preference for oligosaccharides, a substrate for the growth of these bacteria [13, 187, 212]. The beneficial effects of bifidobacteria include the increased absorption of certain nutritional essential minerals, such as calcium, phosphorus, iron, and B vitamins' synthesis [13, 213]. They can also inhibit the binding of pathogens to cell surfaces, thereby preventing some bacterial pathogens' adhesion implicated in chronic infant diarrhea, a leading cause of childhood mortality worldwide [207].

Infant formula manufacturers have proposed two approaches to achieve an intestinal flora more similar to that of breast-fed infants: the addition of nondigestible carbohydrates that may act as prebiotics and/or the addition of cultures of probiotic bacteria such as bifidobacteria and lactobacilli, to mimic infant gastrointestinal colonization. The HMOs are complex components; thus, reproducing them faithfully is very difficult. To mimic HMOs' benefits, simpler synthetic prebiotic oligosaccharides were developed to be used as additives in infant formulas. The most used prebiotic oligosaccharides are GOS and/or FOS [211, 213, 214].

Several bacterial strains included in infant formula have been evaluated for their safety and potential beneficial health effects. These include *Bifidobacterium animalis* subsp. *lactis* CNCMI-3446, alone or in combination with either *Streptococcus thermophilus* or with both *S. thermophilus* and *Lactobacillus helveticus*; *L. johnsonii* La1; *B. longum* BL999 plus *L. rhamnosus* LPR; *L. rhamnosus* GG, *L. reuteri* ATCC 55730; *L. salivarius* CECT5713; and *L. fermentum* CECT5716. The evidence demonstrates that the supplementation of formula with probiotics is not associated with adverse outcomes [211, 215].

The committee on Nutrition of the European Society for Paediatric Gastroenterology, Hepatology, and Nutrition performed a systematic review of the evidence related to the infant safety of infant formula consumption supplemented with probiotics and/or prebiotics compared to nonsupplemented formulas. The scientific data explored in this review suggest that the consumption of infant formulas supplemented with prebiotics and/or probiotics, clinically evaluated in healthy term infants, did not raise concerns about microbiological safety, child development, or adverse effects. However, it is noteworthy that the clinical effects and safety should not be extrapolated to other products. Thus, the committee declared that the supplementation of infant formula with prebiotics and/or probiotics is an important field of infant nutrition research; however, more clinical studies with better experimental designs are needed [211].

## 11. Health Benefits to Bioactive Compounds

Human milk provides a range of bioactive compounds that can provide health benefits beyond the basic nutritional value [31]. Scientific evidence has shown that these compounds in human milk can contribute to the short- and long-term beneficial effects on infants' breastfeed. These bioactive compounds involved a large group of components derived from proteins, vitamins, amino acids, lipids, or carbohydrates. Many of these components have synergistic effects, increasing their biological effects [29]. This section will present some clinical studies evidencing the beneficial effects of these compounds on infant health. Controlled experiments in infant humans are challenging to perform. In this way, most experimental studies were performed in vitro or animal models. Among the bioactive compounds present in human milk, the addition of taurine, folic acid, long-chain polyunsaturated fatty acids, prebiotics, and probiotics to infant formulas is already an accomplishment, while others are still in an experimental phase of efficacy and safety. Despite having demonstrated bioactivity in studies with babies, in vitro or animal models, there is still insufficient scientific data to support its incorporation into commercial infant formulas.

### 11.1. Bioactive Proteins

**Figure 3:**
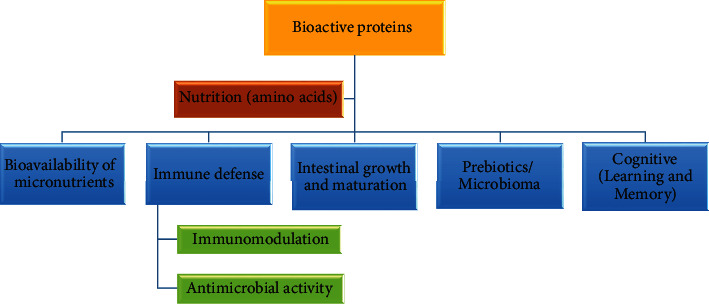
Biological functions of bioactive proteins present in human milk. Adapted from [64].

Bioactive proteins perform many functions (Figure 3), such as a source of amino acids; improve the bioavailability of micronutrients, including vitamins, minerals, and trace elements; support immune defense; antimicrobial activity; stimulate intestinal growth and maturation; and enhance learning and memory [64, 94]. Several of the bioactive proteins' activities are not attributed to the intact proteins but exclusively to released bioactive peptides [42, 43]. Many clinical studies have been developed to evaluate the bioactivities of human milk proteins [45]. Thus, most studies developed to date have used animal models to assess the bioactivity of proteins present in human milk.

Evidence has shown that caseins, both intact and their peptides, serve as sources of amino acids and stimulate mineral absorption. These proteins can also improve different aspects of the immune system, antimicrobial activity, gastrointestinal modulation, antitumor, and opioid-like activity [50–52]. The antimicrobial activity has been reported in different fragments derived from k-casein hydrolyzate [54]. In a study developed by Strömqvist et al. [216], the authors evaluated the effectiveness of cell lineage-specific adhesion of fluoroisothiocyanate-labeled Helicobacter pylori to human gastric surface mucous cells. The results showed that fucose containing carbohydrate moieties of human k-casein are important to inhibit H. pylori adhesion and, therefore, infection. Also, suggesting that breastfeeding can protect against H. pylori infection during early life. In addition to the bioactivity of casein phosphopeptides related to the absorption of calcium, iron, and zinc, studies suggest that these phosphopeptides also have antitumor activity [52]. Shu et al. [217] examined the association between breastfeeding and the development of acute myeloid leukemia and acute lymphoblastic leukemia in two case-control studies carried out by the Children's Cancer Group. Their results demonstrated that breastfeeding was associated with a 21% reduction in the risk of acute leukemia in childhood. A proof-of-concept longitudinal study developed by Gridineva et al. [51] investigated the concentration and daily intake of human milk casein with the anthropometry and body composition of exclusively breastfed infants during the first 12 months of life. This study showed that the daily intake of casein and feeding frequency is associated with the development of the infant's bodily composition and increased fat mass. This result emphasizes the critical role of human milk and breastfeeding in the programming of infant appetite control and growth during the first year of life.

Alpha-lactalbumin is a rich source of essential amino acids, including tryptophan, cysteine, lysine, and branched-chain amino acids (leucine, isoleucine, and valine) all of which are crucial for infant nutrition [45, 67]. In addition, it is also an important protein in the enzyme system of lactose synthesis that can facilitate the absorption of essential minerals, such as calcium, iron, and zinc [27]. Plasma tryptophan concentrations, which are maximal during the night, have been shown to influence newborns' sleep patterns, which is essential for the brain's proper development [218]. The peptides released during the digestion of *α*-lactalbumin have been shown to have antibacterial activities (mostly against gram-positive bacteria and non-gram-negative bacteria) and immunostimulatory properties [27, 43, 219]. Brück and coworkers [220, 221] demonstrated through different in vitro and in vivo studies that *α*-lactalbumin can inhibit several potential pathogens' growth. Pellegrini et al. [219] showed that polypeptide fragments from *α*-lactalbumin exerted bactericidal activity against *E. coli*, *Klebsiella pneumoniae*, *Staphylococcus aureus*, *Staphylococcus epidermis*, *Streptococci*, and *C. Albicans*. Specific peptides arising from the digestion of *α*-lactalbumin have also been shown to encourage bifidobacteria's growth, a species that dominates the gut of breastfed infants [222]. Another physiological characteristic discovered about *α*-La is its tumoricidal activity. Alpha-lactalbumin induces apoptosis in certain cancer cells and bacterial cell death [223] through the complex termed HAMLET (human alpha-lactalbumin made lethal to tumor cells). This complex is formed between alpha-lactalbumin and oleic acid. Evidence from mouse and human trials has shown that HAMLET may potentially have value in the future treatment of several cancers [224]. Another study used a rat model to investigate the use of HAMLET extracted from human milk as a specific treatment for brain tumors. Research focusing on the structure and function of HAMLET continues, intending to develop clinical applications [225].

The main bioactivities of lactoferrin are related to the digestion and absorption of some nutrients such as iron, conferring the newborn's protection against anemia, stimulating the proliferation and differentiation of intestinal cells, and act in cognitive development, improving learning and memory [79, 226]. The antimicrobial properties of lactoferrin are related to the ability to sequester iron from biological fluids, inhibiting the growth of bacteria (gram-positive and gram-negative) that need this nutrient [79]. Another bioactive property of lactoferrin is its potential to interact with the bacterial membrane of pathogens, causing fatal damage against bacterial growth. The interaction of lactoferrin with the bacterial membrane also potentiates the action of other antibacterial factors, such as lysozyme [227]. In addition to the bioactivity of intact lactoferrin, studies have shown that some peptides (lactoferricin) formed from lactoferrin also have potent activity against gram-positive and gram-negative pathogenic bacteria [79]. Hagiwara et al. [228] demonstrated in your experiment that both bovine and human lactoferrin could promote cell proliferation from the gastrointestinal tract. In an in vitro study developed by Buccigrossi et al. [229] found that lactoferrin at higher concentrations induced a strong and rapid increase in intestinal epithelial cell proliferation, whereas low lactoferrin concentrations induced stimulation of intestinal differentiation. These findings suggest that the concentration of lactoferrin is a key modulator of intestinal epithelium development.

Colonization of the intestine with beneficial bacteria is essential for infants' health and well-being [230]. The antimicrobial activity of lactoferrin exerts a beneficial effect on the intestinal microbiota because its bacteriostatic action does not impair these beneficial bacteria's growth, such as bifidobacteria [79]. Liepke et al. [231] demonstrated in their work that the proteolytic fragments derived from lactoferrin stimulate the growth of bifidobacteria and act as prebiotic growth factors. The occurrence of bifidobacteria in the large intestine is beneficial for infants, as it prevents the proliferation of pathogens that cause diarrhea, such as salmonella or rotavirus [230]. The potential role in the neurodevelopment and cognition of lactoferrins has not yet been fully elucidated. Chen et al. [232], to test the hypothesis that lactoferrin can improve neurodevelopment, cognition, and memory, a group of young pigs was supplemented with lactoferrin and the other group not. In terms of cognitive development, piglets supplemented with lactoferrin exhibited improved learning and memory compared with piglets unsupplemented.

Lysozyme, like lactoferrin, exerts antimicrobial activity in the gut of breastfed infants, thus contributing to the development of beneficial intestinal microbiota. Together with lactoferrin, lysozyme can act to kill gram-negative bacteria and also can, regardless of lactoferrin, degrading the outer cell wall of gram-positive bacteria [40, 222]. To determine if lysozyme can modulate gut microbiota composition, Maga and colleagues [88] conducted a study feeding trials in young pigs, followed by a more in-depth fecal microbiota assessment. The authors found that lysozyme modulated gastrointestinal microbiota by increasing the ratio of beneficial bacteria and decreasing disease-causing microbe numbers.

Human milk provides newborn immunomodulatory components (sIgA, IgG, and IgM) that ensure the protection and proper development of the immune system. Secretory IgA antibodies represent the first defense line against several pathogens [93, 222, 233]. Pribylova et al. [233] suggest that the broad spectrum of natural autoantibodies and the high diversity of sIgA present in human milk may contribute proper development of the mucosal immune system of the breastfed infant, exerting anti-inflammatory and tissue-protective activities. SIgA acts by preventing pathogens' access to the intestinal epithelium through a series of processes that involve agglutination, entrapment in mucus, and release by peristaltic movements [94]. Rogier and coworkers [234] developed an experimental system developed in mice to study the long-term benefits of early exposure to IgA secretory antibodies in human milk. Their findings found that human milk-derived SIgA promoted intestinal epithelial barrier function in suckling neonates, preventing systemic infection by potential pathogens. In the long term, early exposure to SIgA could guarantee the maintenance of a healthy gut microbiota, which persists until adulthood [234].

### 11.2. Taurine

Taurine is a nutritionally essential amino acid for children, especially for premature infants [235]. In an infant, taurine performs a wide variety of functions in the central nervous system, from development to neuroprotection [236]. Primary studies demonstrated elevated concentrations of taurine were found in newborn and neonatal brain [237]. Sturman et al. [238] found that when radiolabelled taurine was injected into the peritoneum of a lactating rat, it passed into the milk and accumulated in the brain of the suckling pups, suggesting that this route might be an important source for the central nervous system of taurine. The high concentration of taurine in the developing brain and retina raised the hypothesis that taurine plays an essential role in brain development [239]. It was found that children who had low plasma concentrations of taurine for a long period had abnormalities of the retina. However, both low plasma values and retinal changes were corrected by adding taurine to the diet, suggesting that taurine's adequate intake was important for maintaining brain taurine content [240, 241]. The results obtained by Wharton et al. [242] support the hypothesis that low taurine status in the neonatal period of preterm infants adversely influences later neurodevelopment. These results confirm the view that taurine is a conditionally essential nutrient as a food supply [242].

### 11.3. Folates

Folate is considered an essential micronutrient for humans. This vitamin's physiological need is even critical in the early years of life since this is a period of rapid growth and development. Folates play an essential role in DNA and RNA's biosynthesis and the metabolism of some amino acids, making them essential in cell division and growth. The folate deficiency is initially manifested in the fastest growing tissues, such as bone marrow (erythropoiesis) and mucosa of the gastrointestinal tract [243]. In this way, folate deficiency in infants can cause many unwanted health problems, such as megaloblastic anemia, leukopenia, thrombocytopenia, delayed or abnormal infant development, alteration of the central nervous system maturation, and atrophy of the intestinal villi [24, 110, 244–246]. In addition, poor folate status has also been shown to be a risk factor for respiratory infections in young children [247]. Breastfeed infant is protected from folate deficiency because human milk folate content is maintained at the expense of maternal reserves [248]. Human milk folates have higher bioavailability when compared to folates present in infant formulas because of the folate-binding protein available in human milk, which may facilitate absorption from the gastrointestinal tract [89, 249]. Infant homocysteine metabolism may be regulated through maternal folate concentrations during pregnancy and lactation [248]. In a longitudinal study, Hay et al. [250] measured the folate in serum through total homocysteine at birth and 6, 12, and 24 months of age to determine how breastfeeding and weaning can affect folate status. The authors observed that serum folate concentrations increased from birth to age six months and were declining until age 24 months. Comparing breastfed and nonbreastfed groups, the exclusively breastfed infants had the highest concentrations of serum folate. Donangelo et al. [251] and Foged et al. [252] demonstrated in their studies that infants who were fed with human milk from mothers supplemented or not with folic acid during lactation showed similar blood levels of folate. According to O'Connor et al. [253], when supplemental folic acid is given to mothers with blood folate levels within an acceptable range, an increase in maternal serum folate levels is observed without a concomitant increase in human milk folate concentrations, suggesting that folate intake does not affect milk folate concentration unless a maternal deficiency is severe. Veena et al. [246] reported that higher maternal folate concentration was associated with better cognitive performance in the children. Most of the mothers in this study manifested blood folate levels within the normal range.

### 11.4. Polyamines

Polyamines' requirement is highest when tissues are growing rapidly, which occurs in the early stages of life. Human milk is the first and only source of exogenous polyamines for infants [118, 119, 123, 124]. It is reported that polyamines present in human milk may be beneficial compounds to gut maturation and development and capable of preventing food allergies in breastfed infants [128, 129]. Sabater-Molina et al. [128] evaluated the effects of neonatal milk formulas supplemented with polyamines at the physiologic levels found in maternal milk on early-weaned piglets' gut development. This experiment revealed that polyamines' oral administration at physiologic doses improve the small intestine's development and growth. Fang et al. [254] also obtained similar results.

Regarding the protective effect of human milk against allergies, it has been proposed that milk spermine or spermidine can control food allergy development in neonates. Peulen and colleagues [255] evaluated the correlation between the concentration of polyamine (spermine) in milk intake during the first month after birth and the appearance of allergy in children who ingested this milk. The results showed a clear dependence on the allergy appearance with the polyamine concentration of human milk ingested during the first months after birth. According to the authors' explanations, this is due to the increased maturation of the small intestine and the immune system. This increased leads to a decrease in permeability, which decreases the transfer of antigens from the intestinal lumen to blood circulation [255]. It can be concluded that the polyamines of human milk act only as a preventive agent that regulates or induces the maturation of the intestine, which consequently decreases the allergic reactions caused by food. This behavior explains why allergic problems are less frequent in breastfed infants when compared with not breastfed infants [118, 124, 131].

### 11.5. MFGM

The MFGM that comprise fat droplets responsible for lipid transport in human milk contains several biologically active components. The MFGM has an essential effect on the brain, intestine, and other regions of newborn and infant organimos who receive breast milk. Increasing evidence suggests that the structure of MFGM and its bioactive components may benefit the child by assisting in the structural and functional maturation of the intestine by providing essential nutrients and/or regulating various cellular events during infant growth and immunological education. Also, antimicrobial peptides and fractions of surface carbohydrates around the MFGM can play a key role in the formation of intestinal microbiota composition, which in turn can promote protection against immune and inflammatory diseases early in life [132, 143, 154–156]. Although the underlying mechanism is not entirely clear, MFGM houses two forms of glycoconjugates (glycoproteins and glycolipids), which are believed to have antimicrobial, anti-inflammatory, and prebiotic functions in the intestine. These functions may be responsible for the modulation of the immune and microbiota response. The MFGM is also involved in the development of the central nervous system and its metabolism, supporting brain development and its cognitive functions [149, 152].

#### 11.5.1. LC-PUFAs

The lipids present in human milk, in addition to meeting the infant's high energy needs, are essential for growth, development, and future health [177]. Human milk contains a wide variety of lipid components. Some of them are essentials, such as PUFAs, LC-PUFAs, and fat-soluble vitamins (vitamins A, D, E, and K) [177]. The composition of human milk PUFAs (LA and ALA) and LC-PUFAs (DHA, ARA, and EPA) is markedly modified by maternal dietary habits [139]. Both LA and ALA can influence metabolic processes, such as lowering plasma cholesterol. In addition, they are precursors of endogenous synthesis of the respective LC-PUFAs [177, 256]. Many of the biological effects of the essential fatty acids in early life appear to be mediated by LC-PUFA, which are critical for neurodevelopment, especially for the maturation and maintenance of the brain and retina and for neurological functions [162, 163]. Brain lipids are rich in LC-PUFAs and play a key role in neuronal growth, signal transduction and excitability of neural membranes, and in the expression of genes that regulate cell differentiation and growth [257]. The DHA has shown that it influences the development of executive functions and other higher-order cognitive abilities and has influenced the development of attention and information processing in later childhood [258]. Innis et al. [259] developed a study with exclusively breastfed children for at least three months. Plasma fatty acid concentrations at two months of age; visual acuity at 2, 4, 6, and 12 months; language development at nine months; and mental and psychomotor development index at 6 and 12 months were analyzed. The authors reported a positive correlation of DHA with measures of visual acuity and progression in language development. These results were similar to other clinical studies in which DHA has also been shown to increase visual acuity, motor skills, and language development in premature children [260, 261]. It has also been suggested that LC-PUFAs in human milk can protect against allergy and infection and mediate beneficial long-term effects of breastfeeding on development in cardiometabolic factors such as blood pressure and blood lipid profile [163, 256].

### 11.6. Prebiotic and Probiotic

It is well known that a balanced and diverse microbiota from early childhood is essential to prevent diseases in the short and long term and promote healthy growth and development [262–264]. The feeding practice is an important factor of initial colonization, and clinical studies have already shown that the composition of the intestinal microbiota of infants fed exclusively with human milk is different from those fed with infant formula since prebiotics are not present or not in the same amount [191, 203, 210, 264]. Human milk is rich in prebiotics such as HMO that influence the composition of the intestinal microbiota. HMO promotes commensal bacteria growth, particularly bifidobacteria (*Bifidobacterium infantis*), and growth factors, such as cytokines and immunoglobulins that exert immunomodulatory effects [13, 265]. Furthermore, human milk is a source of bioactive bacteria that can contribute to neonatal gastrointestinal colonization and immune development and maturation. MiSeq sequencing of human milk performed by Murphy et al. [264] revealed a large microbial diversity of the human milk, identifying over 207 bacterial genera in milk samples. However, the predominant bacterial groups were the genera *Lactobacillus*, *Bifidobacterium*, *Pseudomonas*, *Staphylococcus*, *Streptococcus*, and *Enterococcus*. The composition of the fecal microbiota of the breastfeed infants reflects that found in human milk, supporting the notion of vertical transfer via human milk. Breastfeeding is associated with a lower incidence of digestive tract diseases, such as necrotizing enterocolitis and diarrhea and a lower incidence of inflammatory bowel diseases, type 2 diabetes, and obesity later in life [263, 266]. The study carried out by Chichlowski et al. [267] provided evidence to the relation between HMO-grown bifidobacteria and the potentially induce an anti-inflammatory response in the intestinal epithelial cells. A randomized, controlled trial using a probiotic combination (VSL#3) in children with ulcerative colitis has demonstrated the safety and efficacy of this preparation in maintaining disease remission compared with placebo [268, 269]. In children with irritable bowel syndrome, a crossover trial reported that treatment with the same prebiotic combination improved subjective symptoms and reduced abdominal pain and discomfort or bloating and gas compared with placebo [270]. Necrotizing enterocolitis, caused by altered microbial colonization, is a serious disease that affects preterm infants and has serious morbidity and a high mortality rate [271]. Altered microbial colonization, formula feeding, and neonatal stress are thought to be involved in its pathogenesis. Different studies have shown that supplementation with probiotics in preterm infants significantly reduces the incidence of necrotizing enterocolitis and mortality [272–274]. Gut colonization of preterm infants is delayed, which increases the risk of colonization with pathogens compared with full-term newborns [271]. Oral supplementation with lactoferrin alone or in combination with *L. rhamnosus GG* significantly reduces sepsis development in preterm neonates [86].

## 12. Final Considerations

It is essential to make sure that the purpose of infant formula feeding is not to mimic human milk, which is qualitatively incomparable, but to approximate the nutritional characteristics that human milk offers. There is irrefutable evidence that, given the impossibility of breastfeeding, infant formulas are the best alternative for ensuring adequate nutrition. Although the infant food industry has advanced concerning the adequacy of basic nutritional compounds and the inclusion of bioactive compounds, there is no consensus if these novel bioactive ingredients added to infant formulas have the same functional effects found in human milk. Moreover, further studies are needed to assess the bioactivity and bioavailability of these compounds incorporated into infant formulas after the stage of thermal processing and storage since they may lose their bioactivity or decrease their bioavailability. In addition, additional studies should clinically analyze the bioactive compounds already incorporated in infant formulas currently produced and the compounds that are still in the experimental phase to assess their physiological efficacy when added to infant formula.

## Figures and Tables

**Table 1:** Human milk proteins and their biological functions.

Biological function	Bioactive protein
Nutrition	*α*-CN
	*β*-CN
	*κ*-CN
	*α*-La
Digestion and absorption of nutrients	*β*-CN
	*α*-La
	*α*-Antitrypsin
	Antichymotrypsin
Enzymes	Amylase
	Lipase stimulated by bile salt *α*- *α*-La (calcium and zinc)
Nutrient carrier proteins	*β*-CN (calcium, zinc, and phosphorus)
	Folate-binding protein (folate)
	Haptocorrin (B12 vitamin)
	Lf (iron)
Intestinal development	Growth factors
	Lf
Immune defense	*α*-La
	*κ*-CN
	Lf
	Cytokines
	Haptocorrin
	Lactoperoxidase
	Lysozyme
	Osteopontin
	Secretory IgA IgM, IgG, IgD, and IgE
Prebiotic	*α*-La
	Lf
	Glycans
	MFGM proteins^(1)^
Cognitive development	Lf
	MFGM proteins^(1)^

MFGM proteins^(1)^: some bioactive proteins (mucin-1, butyrophilin, CD36, adipophilin, and lactadherin) that are inserted in the milk fat globule membrane (MFGM). CN: casein; La: *α*-lactalbumin; Lf: lactoferrin; Ig: immunoglobulin. Source: Ballard and Morrow [29], Donovan [64], and Haschke et al. [94].

**Table 2:** Average contents (nmol/mL) of polyamines in term human milk and infant formulas.

Polyamines	Human milk	Starting infant formula	Follow-up infant formula
Spermidine	0.124-4.578	0.186-6.933	0.138-4.241
Spermine	0.104-5.080	0.129-7.339	0.158-6.227
Putrescine	0.030-896	0.018-14.300	0.263-12.796

Source: Muñoz-Esparza et al. [122].

## Data Availability

The data that supports the findings of this study are available upon request.

## Supplementary Materials

Supplementary Materials Supplementary materials can be found at (link to manuscript tracking). Table S1: biological functions of bioactive compounds and recommendations to marketed infant formulas (regulated and unregulated compounds). Table S2: main clinical findings related to the enrichment of infant formulas with bioactive compounds and their effects on infant health.

## References

[1] Lanigan J., Singhal A. (2009). Early nutrition and long-term health: a practical approach. *Proceedings of the Nutrition Society*.

[2] WHO (2014). Global nutrition targets 2025: breastfeeding policy brief.

[3] Global Nutrition Report (2018). The 2018 Global Nutrition Report is shining a light to spur action on nutrition.

[4] United Nations Children’s Fund (2014). UNICEF’s Approach to Scaling Up Nutrition for Mothers and Their Children. *Programme Division, UNICEF*.

[5] Victora C. G., Bahl R., Barros A. J. D. (2016). Breastfeeding in the 21st century: epidemiology, mechanisms, and lifelong effect. *The Lancet*.

[6] EFSA European Food Safety Authority (2014). Scientific opinion on the essential composition of infant and follow-on formulae. *EFSA Journal*.

[7] Nucci A. M., Virtanen S. M., Becker D. J. (2015). Infant feeding and timing of complementary foods in the development of type 1 diabetes. *Current Diabetes Reports*.

[8] Owen C. G., Martin R. M., Whincup P. H., Smith G. D., Cook D. G. (2006). Does breastfeeding influence risk of type 2 diabetes in later life? A quantitative analysis of published evidence. *The American Journal of Clinical Nutrition*.

[9] Gabrielli O., Zampini L., Galeazzi T. (2011). Preterm milk oligosaccharides during the first month of lactation. *Pediatrics*.

[10] Belderbos M. E., Houben M. L., van Bleek G. M. (2012). Breastfeeding modulates neonatal innate immune responses: a prospective birth cohort study. *Pediatric Allergy and Immunology*.

[11] Vandenplas Y. (2017). Prevention and management of cow’s milk allergy in non-exclusively breastfed infants. *Nutrients*.

[12] Maisels M. J. (2006). Neonatal jaundice. *Pediatrics in Review*.

[13] Thomson P., Medina D. A., Garrido D. (2018). Human milk oligosaccharides and infant gut bifidobacteria: molecular strategies for their utilization. *Food Microbiology*.

[14] van den Broek L. A. M., Hinz S. W. A., Beldman G., Vincken J. P., Voragen A. G. J. (2008). Bifidobacterium carbohydrases – their role in breakdown and synthesis of (potential) prebiotics. *Molecular Nutrition & Food Research*.

[15] Comité de nutrition de la Société française de pédiatrie, Turck D., Vidailhet M. (2013). Breastfeeding: health benefits for child and mother. *Archives de Pédiatrie*.

[16] Martins M. Z. (2013). Benefícios da amamentação para saúde materna. *Interfaces Científicas - Saúde e Ambiente*.

[17] EFSA Panel on Nutrition, Novel Foods and Food Allergens, Castenmiller J., de Henauw S. (2019). Appropriate age range for introduction of complementary feeding into an infant’s diet. *EFSA Journal*.

[18] EFSA, European Commission (2006). *Commission Directive 2006/141/EC of 22 December 2006 on infant formulae and follow-on formulae and amending Directive 1999/21/EC*.

[19] World Health Organization & UNICEF (2003). *Global Strategy for Infant and Young Children*.

[20] American Academy of Pediatrics (2012). Breastfeeding and the use of human milk. *Pediatrics*.

[21] Committee on Drugs, American Academy of Pediatrics (2001). The transfer of drugs and other chemicals into human milk. *Pediatrics*.

[22] WHO (2010). *Guidelines on HIV and Infant Feeding*.

[23] Seoane R. G., Garcia-Recio V., Garrosa M. (2020). Human health effects of lactose consumption as a food and drug ingredient. *Current Pharmaceutical Design*.

[24] Sociedade Brasileira de Pediatria(SPB) (2018). Departamento de Nutrologia. *Manual de Alimentação: Orientações para Alimentação do Lactente ao Adolescente, Na Escola, Na Gestante, Na Prevenção de Doenças e Segurança Alimentar*.

[25] Martin C. R., Ling P.-R., Blackburn G. L. (2016). Review of infant feeding: key features of breast milk and infant formula. *Nutrients*.

[26] Koletzko B., Shamir R., Ashwell M. (2012). Quality and safety aspects of infant nutrition. *Animal of Nutricion & Metabolism*.

[27] Lönnerdal B. (2014). Infant formula and infant nutrition: bioactive proteins of human milk and implications for composition of infant formulas. *The American Journal of Clinical Nutrition*.

[28] Lönnerdal B. L., Hernell O. (2016). An opinion on “staging” of infant formula: a developmental perspective on infant feeding. *Journal of Pediatric Gastroenterology and Nutrition*.

[29] Ballard O., Morrow A. L. (2013). Human milk composition: nutrients and bioactive factors. *Pediatric Clinics of North America*.

[30] Park Y. W., Nam M. S. (2015). Bioactive peptides in milk and dairy products: a review. *Korean Journal for Food Science of Animal Resources*.

[31] Biesalski H.-K., Dragsted L. O., Elmadfa I. (2009). Bioactive compounds: definition and assessment of activity. *Nutrition*.

[32] Ahern G. J., Hennessy A. A., Anthony Ryan C., Paul Ross R., Stanton C. (2019). Advances in infant formula science. *Annual Review of Food Science and Technology*.

[33] Bravi F., Wiens F., Decarli A., Pont A. D., Agostoni C., Ferraroni M. (2016). Impact of maternal nutrition on breast-milk composition: a systematic review. *The American Journal of Clinical Nutrition*.

[34] Meisel H., Fitz Gerald R. J. (2003). Biofunctional peptides from milk proteins: mineral binding and cytomodulatory effects. *Current Pharmaceutical Design*.

[35] Hernell O. (2011). Human milk vs. cow’s milk and the evolution of infant formulas. *Milk and Milk Products in Human Nutrition*.

[36] Rêgo C., Pereira-da-silva L., Ferreira R. (2018). CoFI - Consenso sobre Fórmulas Infantis: a opinião de peritos portugueses sobre a sua composição e indicações. *Acta Médica Portuguesa*.

[37] FDA Food and Drug Administration (2014). Current good manufacturing practices, quality control procedures, quality factors, notification requirements, and records and reports, for infant formula. *Regulatory impact analysis for final rule. Federal Register. United States*.

[38] Lönnerdal B. (2012). Preclinical assessment of infant formula. *Annals of Nutrition and Metabolism*.

[39] Moher D., Liberati A., Tetzlaff J., Altman D. G., The PRISMA Group (2009). Preferred reporting items for systematic reviews and meta-analyses: the PRISMA statement. *PLoS Medicine*.

[40] Golinelli L. P., Del Aguila E. M., Flosi Paschoalin V. M., Silva J. T., Conte-Junior C. A. (2014). Functional aspect of colostrum and whey proteins in human milk. *Journal of Human Nutrition & Food Science*.

[41] Brück W. M., Kelleher S. L., Gibson G. R., Graverholt G., Lönnerdal B. L. (2006). The effects of alpha-lactalbumin and glycomacropeptide on the association of CaCo-2 cells by enteropathogenic Escherichia coli, Salmonella typhimurium and Shigella flexneri. *FEMS Microbiology Ecology*.

[42] Liao Y., Jiang R., Lönnerdal B. (2012). Biochemical and molecular impacts of lactoferrin on small intestinal growth and development during early life. *Biochemistry and Cell Biology*.

[43] Wada Y., Lönnerdal B. (2014). Bioactive peptides derived from human milk proteins -- mechanisms of action. *The Journal of Nutritional Biochemistry*.

[44] Dallas D. C., Guerrero A., Khaldi N. (2013). Extensive in vivo human milk peptidomics reveals specific proteolysis yielding protective antimicrobial peptides. *Journal of Proteome Research*.

[45] Lönnerdal B. (2017). Bioactive proteins in human milk--potential benefits for preterm infants. *Clinics in Perinatology*.

[46] Khaldi N., Vijayakumar V., Dallas D. C. (2014). Predicting the important enzymes in human breast milk digestion. *Journal of Agricultural and Food Chemistry*.

[47] Holton T. A., Vijayakumar V., Dallas D. C. (2014). Following the digestion of milk proteins from mother to baby. *Journal of Proteome Research*.

[48] Lönnerdal B. (2016). Bioactive proteins in human milk: health, nutrition, and implications for infant formulas. *The Journal of Pediatrics*.

[49] Almeida C. C., Conte-Júnior C. A., Silva A. C. O., Alvares T. S. (2013). Whey protein: composition and functional properties. *Enciclopédia Biosfera*.

[50] Enjapoori A. K., Kukuljan S., Dwyer K. M., Sharp J. A. (2019). In vivo endogenous proteolysis yielding beta-casein derived bioactive beta- casomorphin peptides in human breast milk for infant nutrition. *Nutrition*.

[51] Gridneva Z., Tie W. J., Rea A. (2018). Human milk casein and whey protein and infant body composition over the first 12 months of lactation. *Nutrients*.

[52] Ledesma-Martínez E., Aguíñiga-Sánchez I., Weiss-Steider B., Rivera-Martínez A. R., Santiago-Osorio E. (2019). Casein and peptides derived from casein as antileukaemic agents. *Journal of Oncology*.

[53] Hebert E. M., Saavedra L., Ferranti P., Mozzi F., Raya R. R., Vignolo G. M. (2010). Bioactive peptides derived from casein and whey proteins. *Biotechnology of Lactic Acid Bacteria: Novel Applications*.

[54] Hernández-Ledesma B., Quirós A., Amigo L., Recio I. (2007). Identification of bioactive peptides after digestion of human milk and infant formula with pepsin and pancreatin. *International Dairy Journal*.

[55] Sánchez A., Vázquez A. (2017). Bioactive peptides: a review. *Food Quality and Safety*.

[56] Brück W. M., Kelleher S. L., Gibson G. R., Nielsen K. E., Chatterton D. E. W., Lönnerdal B. (2003). rRNA probes used to quantify the effects of glycomacropeptide and alpha-lactalbumin supplementation on the predominant groups of intestinal bacteria of infant rhesus monkeys challenged with enteropathogenic Escherichia coli. *Journal of Pediatric Gastroenterology and Nutrition*.

[57] Miquel E., Alegría A., Barberá R., Farré R. (2006). Casein phosphopeptides released by simulated gastrointestinal digestion of infant formulas and their potential role in mineral binding. *International Dairy Journal*.

[58] Jarmołowska B., Sidor K., Iwan M. (2007). Changes of *β*-casomorphin content in human milk during lactation. *Peptides*.

[59] Kamiński S., Cieslińska A., Kostyra E. (2007). Polymorphism of bovine beta-casein and its potential effect on human health. *Journal of Applied Genetics*.

[60] Cattaneo S., Pica V., Stuknytė M. (2020). Effect of protein fortification on heat damage and occurrence of *β*-casomorphins in (un)digested donor human milk intended for nutrition of preterm infants. *Food Chemistry*.

[61] EFSA, European Food Safety Authority (2009). Review of the potential health impact of *β*-casomorphins and related peptides. *EFSA Journal*.

[62] Jarmołowska B., Szłapka-Sienkiewicz E., Kostyra E., Kostyra H., Mierzejewska D., Marcinkiewicz-Darmochwał K. (2007). Opioid activity of Humana formula for newborns. *Journal of the Science of Food and Agriculture*.

[63] Davis A., Harris B., Lien E., Pramuk K., Trabulsi J. (2008). *α* -Lactalbumin-rich infant formula fed to healthy term infants in a multicenter study: plasma essential amino acids and gastrointestinal tolerance. *European Journal of Clinical Nutrition*.

[64] Donovan S. M., Donovan S. M., German J. B., Lönnerdal B., Lucas A. (2019). Human milk proteins: composition and physiological significance. *Human Milk: Composition, Clinical Benefits and Future Opportunities*.

[65] Solé D., Silva L. R., Cocco R. R. (2018). Consenso Brasileiro sobre Alergia Alimentar: 2018 - parte 2 - Diagnóstico, tratamento e prevenção. Documento conjunto elaborado pela Sociedade Brasileira de Pediatria e Associação Brasileira de Alergia e Imunologia. *Arquivos de Asma, Alergia e Imunologia*.

[66] Pastor N., Soler B., Mitmesser S. H., Ferguson P., Lifschitz C. (2006). Infants fed docosahexaenoic acid- and arachidonic acid-supplemented formula have decreased incidence of bronchiolitis/bronchitis the first year of life. *Clinical Pediatrics*.

[67] Layman D. K., Lönnerdal B., Fernstrom J. D. (2018). Applications for *α*-lactalbumin in human nutrition. *Nutrition Reviews*.

[68] Sandström O., Lönnerdal B., Graverholt G., Hernell O. (2008). Effects of *α*-lactalbumin–enriched formula containing different concentrations of glycomacropeptide on infant nutrition. *The American Journal of Clinical Nutrition*.

[69] Kamau S. M., Cheison S. C., Chen W., Liu X.-M., Lu R.-R. (2010). Alpha-lactalbumin: its production technologies and bioactive peptides. *Comprehensive Reviews in Food Science and Food Safety*.

[70] Lönnerdal B., Lien E. L. (2003). Nutritional and physiologic significance of *α*-lactalbumin in infants. *Nutrition Reviews*.

[71] Trabulsi J., Capeding R., Lebumfacil J. (2011). Effect of an *α*-lactalbumin-enriched infant formula with lower protein on growth. *European journal of clinical nutrition*.

[72] Fleddermann M., Demmelmair H., Grote V., Nikolic T., Trisic B., Koletzko B. (2014). Infant formula composition affects energetic efficiency for growth: the BeMIM study, a randomized controlled trial. *Clinical Nutrition (Edinburgh, Scotland)*.

[73] Szymlek-Gay E. A., Lonnerdal B., Abrams S. A., Kvistgaard A. S., Domellof M., Hernell O. (2012). *α*-Lactalbumin and casein-glycomacropeptide do not affect iron absorption from formula in healthy term infants. *The Journal of Nutrition*.

[74] Johnston W. H., Ashley C., Yeiser M. (2015). Growth and tolerance of formula with lactoferrin in infants through one year of age: double-blind, randomized, controlled trial. *BMC Pediatrics*.

[75] Yang Z., Jiang R., Chen Q. (2018). Concentration of lactoferrin in human milk and its variation during lactation in different chinese populations. *Nutrients*.

[76] Lönnerdal B. (2009). Nutritional roles of lactoferrin. *Current Opinion in Clinical Nutrition and Metabolic Care*.

[77] Telang S. (2018). Lactoferrin: a critical player in neonatal host defense. *Nutrients*.

[78] Pai U., Chandrasekhar P., Carvalho R. S., Kumar S. (2018). The role of nutrition in immunity in infants and toddlers: an expert panel opinion. *Clinical Epidemiology and Global Health*.

[79] de Queiroz V. A., Assis A. M., Júnior H. d. C. (2013). Efeito protetor da lactoferrina humana no trato gastrintestinal. *Revista Paulista de Pediatria*.

[80] Vogel H. J. (2012). Lactoferrin, a bird’s eye view. *Biochemistry and Cell Biology*.

[81] Aly E. G., Ros G., Frontela C. (2013). Structure and functions of lactoferrin as ingredient in infant formulas. *Journal of Food Research*.

[82] King J. C., Cummings G. E., Guo N. (2007). A double-blind, placebo-controlled, pilot study of bovine lactoferrin supplementation in bottle-fed infants. *Journal of Pediatric Gastroenterology and Nutrition*.

[83] Lönnerdal B., Jiang R., Du X. (2011). Bovine lactoferrin can be taken up by the human intestinal lactoferrin receptor and exert bioactivities. *Journal of Pediatric Gastroenterology and Nutrition*.

[84] Gill B. D., Indyk H. E., Woollard D. C. (2016). Current methods for the analysis of selected novel nutrients in infant formulas and adult nutritionals. *Journal of AOAC International*.

[85] Zemankova N., Chlebova K., Matiasovic J., Prodelalova J., Gebauer J., Faldyna M. (2016). Bovine lactoferrin free of lipopolysaccharide can induce a proinflammatory response of macrophages. *BMC veterinary research*.

[86] Manzoni P., Rinaldi M., Cattani S. (2009). Bovine lactoferrin supplementation for prevention of late-onset sepsis in very low-birth-weight neonates: a randomized trial. *JAMA*.

[87] O’Callaghan D. M., O’Mahony J. A., Ramanujam K. S., Burgher A. M. (2011). Dehydrated dairy products | infant formulae. *Encyclopedia of Dairy Sciences*.

[88] Maga E. A., Desai P. T., Weimer B. C., Dao N., Kültz D., Murray J. D. (2012). Consumption of lysozyme-rich milk can alter microbial fecal populations. *Applied and Environmental Microbiology*.

[89] Hendricks G. M., Guo M., Guo M. (2014). Bioactive components in human milk. *Human Milk Biochemistry and Infant Formula Manufacturing Technology*.

[90] Kaiser G. G., Mucci N. C., González V. (2017). Detection of recombinant human lactoferrin and lysozyme produced in a bitransgenic cow. *Journal of Dairy Science*.

[91] Hurley W. L., Theil P. K. (2011). Perspectives on immunoglobulins in colostrum and milk. *Nutrients*.

[92] Nguyen T. T. P., Bhandari B., Cichero J., Prakash S. (2015). A comprehensive review on in vitro digestion of infant formula. *Food Research International*.

[93] Basha S., Surendran N., Pichichero M. (2014). Immune responses in neonates. *Expert Review of Clinical Immunology*.

[94] Haschke F., Haiden N., Thakkar S. K. (2017). Nutritive and bioactive proteins in breastmilk. *Annals of Nutrition and Metabolism*.

[95] Roysommuti S., Wyss J. M. (2014). Perinatal taurine exposure affects adult arterial pressure control. *Amino Acids*.

[96] Manzi P., Pizzoferrato L. (2013). Taurine in milk and yoghurt marketed in Italy. *International Journal of Food Sciences and Nutrition*.

[97] Bouckenooghe T., Remacle C., Reusens B. (2006). Is taurine a functional nutrient?. *Current Opinion in Clinical Nutrition and Metabolic Care*.

[98] Ripps H., Shen W. (2012). Review: Taurine: a “very essential” amino acid. *Molecular Vision*.

[99] Kilb W., Fukuda A. (2017). Taurine as an essential neuromodulator during perinatal cortical development. *Frontiers in Cellular Neuroscience*.

[100] Yu M., Wang Y., Wang Z., Liu Y., Yu Y., Gao X. (2019). Taurine promotes milk synthesis via the GPR87-PI3K-SETD1A signaling in BMECs. *Journal of Agricultural and Food Chemistry*.

[101] Chuang C.-K., Lin S.-P., Lee H.-C. (2005). Free amino acids in full-term and pre-term human milk and infant formula. *Journal of Pediatric Gastroenterology and Nutrition*.

[102] Codex Alimentarius (2007). Standard for infant formula and formulas for special medical purposes intended for infants. *Adopted as a worldwide Standard in 1981. Amendment: 1983, 1985, 1987, 2011 and 2015. Revision: 2007*.

[103] Verner A., McGuire W., Craig J. S., Cochrane Neonatal Group (2007). Effect of taurine supplementation on growth and development in preterm or low birth weight infants. *The Cochrane database of systematic reviews*.

[104] Cao S., Jiang H., Niu S.-P., Wang X.-H., Du S. (2018). Effects of taurine supplementation on growth in low birth weight infants: a systematic review and meta-analysis. *The Indian Journal of Pediatrics*.

[105] Zhang Z., Adelman A. S., Rai D., Boettcher J., Lőnnerdal B. (2013). Amino acid profiles in term and preterm human milk through lactation: a systematic review. *Nutrients*.

[106] Campos-Giménez E., Bénet S., Oguey Y., Martin F., Redeuil K. (2017). The contribution of minor folates to the total vitamin B9 content of infant formula and clinical nutrition products. *Food Chemistry*.

[107] Yaman M., Mızrak Ö. F., Çatak J., Sargın H. S. (2019). In vitro bioaccessibility of added folic acid in commercially available baby foods formulated with milk and milk products. *Food Science and Biotechnology*.

[108] Taylor D., May P. (2008). Folic Acid. A necessary ingredient for building DNA, cells and babies. http://www.chm.bris.ac.uk/motm/folic-acid/folicjs.htm.

[109] Czeizel A. E., Dudás I., Vereczkey A., Bánhidy F. (2013). Folate deficiency and folic acid supplementation: the prevention of neural-tube defects and congenital heart defects. *Nutrients*.

[110] Hermoso M., Vollhardt C., Bergmann K., Koletzko B. (2011). Critical micronutrients in pregnancy, lactation, and infancy: considerations on vitamin D, folic acid, and iron, and priorities for future research. *Annals of Nutrition and Metabolism*.

[111] Black M. M. (2008). Effects of vitamin B12 and folate deficiency on brain development in children. *Food and Nutrition Bulletin*.

[112] Ohrvik V. E., Witthoft C. M. (2011). Human folate bioavailability. *Nutrients*.

[113] Lamers Y. (2011). Folate recommendations for pregnancy, lactation, and infancy. *Annals of Nutrition and Metabolism*.

[114] Crider K. S., Bailey L. B., Berry R. J. (2011). Folic acid food fortification-its history, effect, concerns, and future directions. *Nutrients*.

[115] Troesch B., Demmelmair J., Gimpfl M. (2019). Suitability and safety of L-5-methyltetrahydrofolate as a folate source in infant formula: a randomized-controlled trial. *PloS one*.

[116] Smith A. D., Kim Y. I., Refsum H. (2008). Is folic acid good for everyone?. *The American Journal of Clinical Nutrition*.

[117] Ami N., Bernstein M., Boucher F., Rieder M., Parker L. (2016). Folate and neural tube defects: the role of supplements and food fortification. *Paediatrics and Child Health*.

[118] Garwolińska D., Namieśnik J., Kot-Wasik A., Hewelt-Belka W. (2018). Chemistry of human breast milk – a comprehensive review of the composition and role of milk metabolites in child development. *Journal of Agricultural and Food Chemistry*.

[119] Ruiz-Cano L. D., Pérez-Llamas F., Zamora S. (2012). Polyamines, implications for infant health. *Archivos Argentinos de Pediatria*.

[120] Bjelakovic L., Kocic G., Bjelakovic B. (2012). Polyamine oxidase and diamine oxidase activities in human milk during the first month of lactation. *Iranian Journal of Pediatrics*.

[121] Gómez-Gallego C., Collado M. C., Ilo T. (2012). Infant formula supplemented with polyamines alters the intestinal microbiota in neonatal BALB/cOlaHsd mice. *The Journal of Nutritional Biochemistry*.

[122] Muñoz-Esparza N. C., Latorre-Moratalla M. L., Comas-Basté O., Toro-Funes N., Veciana-Nogués M. T., Vidal-Carou M. C. (2019). Polyamines in food. *Frontiers in Nutrition*.

[123] Büyükuslu N. (2015). Polyamines in human breast milk. *Journal of Current Pediatrics*.

[124] Larqué E., Sabater-Molina M., Zamora S. (2007). Biological significance of dietary polyamines. *Nutrition*.

[125] Plaza-Zamora J., Sabater-Molina M., Rodríguez-Palmero M. (2013). Polyamines in human breast milk for preterm and term infants. *British Journal of Nutrition*.

[126] Ali M. A., Strandvik B., Sabel K.-G., Kilander C. P., Strömberg R., Yngve A. (2014). Polyamine levels in breast milk are associated with mothers’ dietary intake and are higher in preterm than full-term human milk and formulas. *Journal of Human Nutrition and Dietetics*.

[127] Gómez-Gallego C., Kumar H., García-Mantrana I. (2017). Breast milk polyamines and microbiota interactions: impact of mode of delivery and geographical location. *Annals of Nutrition and Metabolism*.

[128] Sabater-Molina M., Larqué E., Torrella F. (2009). Effects of dietary polyamines at physiologic doses in early-weaned piglets. *Nutrition*.

[129] Jacobi S. K., Odle J. (2012). Nutritional factors influencing intestinal health of the neonate. *Advances in Nutrition*.

[130] Pérez-Cano F. J., González-Castro A., Castellote C., Franch À., Castell M. (2010). Influence of breast milk polyamines on suckling rat immune system maturation. *Developmental & Comparative Immunology*.

[131] Gómez-Gallego C., Frias R., Pérez Martínez G. (2014). Polyamine supplementation in infant formula: influence on lymphocyte populations and immune system-related gene expression in a Balb/cOlaHsd mouse model. *Food Research International*.

[132] Hernell O., Timby N., Domellöf M., Lönnerdal B. (2016). Clinical benefits of milk fat globule membranes for infants and children. *The Journal of Pediatrics*.

[133] Nieto-Ruiz A., García-Santos J. A., Bermúdez M. G. (2019). Cortical visual evoked potentials and growth in infants fed with bioactive compounds-enriched infant formula: results from COGNIS randomized clinical trial. *Nutrients*.

[134] Ortega-Anaya J., Jiménez-Flores R. (2019). Symposium review: The relevance of bovine milk phospholipids in human nutrition --evidence of the effect on infant gut and brain development. *Journal of Dairy Science*.

[135] Dewettinck K., Rombaut R., Thienpont N., Le T. T., Messens K., Van Camp J. (2008). Nutritional and technological aspects of milk fat globule membrane material. *International Dairy Journal*.

[136] Sánchez-Juanes F., Alonso J. M., Zancada L., Hueso P. (2009). Glycosphingolipids from bovine milk and milk fat globule membranes: a comparative study. Adhesion to enterotoxigenic Escherichia coli strains. *Biological Chemistry*.

[137] Bourlieu C., Bouzerzour K., Ferret-Bernard S. (2015). Infant formula interface and fat source impact on neonatal digestion and gut microbiota. *European Journal of Lipid Science and Technology*.

[138] Tanaka K., Hosozawa M., Kudo N. (2013). The pilot study: sphingomyelin-fortified milk has a positive association with the neurobehavioural development of very low birth weight infants during infancy, randomized control trial. *Brain & Development*.

[139] Koletzko B. (2017). Human milk lipids. *Annals of Nutrition and Metabolism*.

[140] Fontecha J., Brink L., Wu S., Pouliot Y., Visioli F., Jiménez-Flores R. (2020). Sources, production, and clinical treatments of milk fat globule membrane for infant nutrition and well-being. *Nutrients*.

[141] Lopez C., Ménard O. (2011). Human milk fat globules: polar lipid composition and _in situ_ structural investigations revealing the heterogeneous distribution of proteins and the lateral segregation of sphingomyelin in the biological membrane. *Colloids and Surfaces B: Biointerfaces*.

[142] McJarrow P., Schnell N., Jumpsen J., Clandinin T. (2009). Influence of dietary gangliosides on neonatal brain development. *Nutrition Reviews*.

[143] Lee H., Padhi E., Hasegawa Y. (2018). Compositional dynamics of the milk fat globule and its role in infant development. *Frontiers in Pediatrics*.

[144] Cavalleto M., Giuffrida M. G., Conti A., Bösze Z. (2008). Milk fat globule membrane components–a proteomic approach. *Bioactive Components of Milk*.

[145] Manoni M., Di Lorenzo C., Ottoboni M., Tretola M., Pinotti L. (2020). Comparative proteomics of milk fat globule membrane (MFGM) proteome across species and lactation stages and the potentials of MFGM fractions in infant formula preparation. *Foods*.

[146] Cao X., Zheng Y., Wu S., Yang N., Yue X. (2019). Characterization and comparison of milk fat globule membrane N-glycoproteomes from human and bovine colostrum and mature milk. *Food & Function*.

[147] Liao Y., Alvarado R., Phinney B., Lönnerdal B. (2011). Proteomic characterization of human milk fat globule membrane proteins during a 12 month lactation period. *Journal of Proteome Research*.

[148] Lawrence R. A., Lawrence R. M., Lawrence R. A., Lawrence R. M. (2011). Biochemistry of human milk. *Breastfeeding*.

[149] Timby N., Domellöf M., Lönnerdal B., Hernell O. (2017). Supplementation of infant formula with bovine milk fat globule membranes. *Advances in Nutrition: An International Review Journal*.

[150] Timby K., Domellof E., Hernell O., Lonnerdal B., Domellof M. (2014). Neurodevelopment, nutrition, and growth until 12 mo of age in infants fed a low-energy, low-protein formula supplemented with bovine milk fat globule membranes: a randomized controlled trial. *The American Journal of Clinical Nutrition*.

[151] Timby N., Hernell O., Vaarala O., Melin M., Lönnerdal B., Domellöf M. (2015). Infections in infants fed formula supplemented with bovine milk fat globule membranes. *Journal of Pediatric Gastroenterology & Nutrition*.

[152] Gurnida D. A., Rowan A. M., Idjradinata P., Muchtadi D., Sekarwana N. (2012). Association of complex lipids containing gangliosides with cognitive development of 6-month-old infants. *Early Human Development*.

[153] Palmano K., Rowan A., Guillermo R., Guan J., McJarrow P. (2015). The role of gangliosides in neurodevelopment. *Nutrients*.

[154] Li X., Peng Y., Li Z. (2019). Feeding infants formula with probiotics or milk fat globule membrane: a double-blind, randomized controlled trial. *Frontiers in Pediatrics*.

[155] Huërou-Luron I. L., Lemaire M., Blat S. (2018). Health benefits of dairy lipids and MFGM in infant formula. *Oilseeds and Fats, Crops and Lipids*.

[156] Nelly Z., Anne Staudt K., Gitte G. (2011). Efficacy of an MFGM-enriched complementary food in diarrhea, anemia, and micronutrient status in infants. *Journal of Pediatric Gastroenterology and Nutrition*.

[157] Koletzko B., Lien E., Agostoni C. (2008). The roles of long-chain polyunsaturated fatty acids in pregnancy, lactation and infancy: review of current knowledge and consensus recommendations. *Journal of Perinatal Medicine*.

[158] Hageman J. H. J., Danielsen M., Nieuwenhuizen A. G., Feitsma A. L., Dalsgaard T. K. (2019). Comparison of bovine milk fat and vegetable fat for infant formula: implications for infant health. *International Dairy Journal.*.

[159] Lavialle M., Denis I., Guesnet P., Vancassel S. (2010). Involvement of omega-3 fatty acids in emotional responses and hyperactive symptoms. *The Journal of Nutritional Biochemistry*.

[160] Hoffman D. R., Boettcher J. A., Diersen-Schade D. A. (2009). Toward optimizing vision and cognition in term infants by dietary docosahexaenoic and arachidonic acid supplementation: a review of randomized controlled trials. *Prostaglandins, Leukotrienes and Essential Fatty Acids*.

[161] Lien E. L., Richard C., Hoffman D. R. (2018). DHA and ARA addition to infant formula: current status and future research directions. *Prostaglandins, Leukotrienes and Essential Fatty Acids*.

[162] Lapillonne A., Pastor N., Zhuang W., Scalabrin D. M. F. (2014). Infants fed formula with added long chain polyunsaturated fatty acids have reduced incidence of respiratory illnesses and diarrhea during the first year of life. *BMC Pediatrics*.

[163] Tinoco S. M. B., Sichieri R., Moura A. S., da Silva Santos F., das Graças Tavares do Carmo M. (2007). The importance of essential fatty acids and the effect of trans fatty acids in human milk on fetal and neonatal development. *Cadernos de Saúde Pública*.

[164] Echeverría F., Valenzuela R., Hernandez-Rodas M. C., Valenzuela A. (2017). Docosahexaenoic acid (DHA), a fundamental fatty acid for the brain: new dietary sources. *Prostaglandins, Leukotrienes and Essential Fatty Acids*.

[165] Hadley K. B., Ryan A. S., Forsyth S., Gautier S., Salem N. (2016). The essentiality of arachidonic acid in infant development. *Nutrients*.

[166] George A. D., Gay M., Trengove R. D., Geddes D. (2018). Human milk lipidomics: current techniques and methodologies. *Nutrients*.

[167] Weiser M. J., Butt C. M., Mohajeri M. H. (2016). Docosahexaenoic acid and cognition throughout the lifespan. *Nutrients*.

[168] Calder P. C. (2016). Docosahexaenoic acid. *Annals of Nutrition and Metabolism*.

[169] Mena P., Uauy R., Koletzko B. (2015). Nutritional needs fats. *In Pediatric Nutrition in Practice*.

[170] ILSI, International Life Sciences Institute of Brazil (2018). Dinâmica da composição do leite humano e suas implicações clínicas. https://ilsibrasil.org/publication/dinamica-da-composicao-do-leite-humano-e-suas-implicacoes-clinicas/.

[171] Rogers L. K., Valentine C. J., Keim S. A. (2013). DHA supplementation: current implications in pregnancy and childhood. *Pharmacological Research*.

[172] Thompkinson D. K., Kharb S. (2007). Aspects of infant food formulation. *Comprehensive Reviews in Food Science and Food Safety*.

[173] Jasani B., Simmer K., Patole S. K., Rao S. C. (2017). Long chain polyunsaturated fatty acid supplementation in infants born at term. *Cochrane database of systematic reviews*.

[174] Mendonça M. A., Araújo W. M. C., Borgo L. A., de Rodrigues Alencar E. (2017). Lipid profile of different infant formulas for infants. *PloS One*.

[175] Qawasmi A., Landeros-Weisenberger A., Bloch M. H. (2013). Meta-analysis of LCPUFA supplementation of infant formula and visual acuity. *Pediatrics*.

[176] Qawasmi A., Landeros-Weisenberger A., Leckman J. F., Bloch M. H. (2012). Meta-analysis of long-chain polyunsaturated fatty acid supplementation of formula and infant cognition. *Pediatrics*.

[177] Delplanque B., Gibson R., Koletzko B., Lapillonne A., Strandvik B. (2015). Lipid quality in infant nutrition: current knowledge and future opportunities. *Journal of Pediatric Gastroenterology and Nutrition*.

[178] Gianni M. L., Roggero P., Baudry C., Fressange-Mazda C., le Ruyet P., Mosca F. (2018). No effect of adding dairy lipids or long chain polyunsaturated fatty acids on formula tolerance and growth in full term infants: a randomized controlled trial. *BMC Pediatrics*.

[179] WHO (2013). *Review of the Codex Standard for Follow-Up Formula (CODEX STAN 156-1987)*.

[180] Pacheco A. R., Barile D., Underwood M. A., Mills D. A. (2015). The impact of the milk glycobiome on the neonate gut microbiota. *Annual Review of Animal Biosciences*.

[181] Hutkins R. W., Krumbeck J. A., Bindels L. B. (2016). Prebiotics: why definitions matter. *Current Opinion in Biotechnology*.

[182] Boehm G., Stahl B. (2007). Oligosaccharides from milk. *The Journal of Nutrients*.

[183] Elwakiel M., Hageman J. A., Wang W. (2018). Human milk oligosaccharides in colostrum and mature milk of Chinese mothers: Lewis positive secretor subgroups. *Journal of Agricultural and Food Chemistry*.

[184] Smilowitz J. T., Lebrilla C. B., Mills D. A., German J. B., Freeman S. L. (2014). Breast milk oligosaccharides: structure-function relationships in the neonate. *Annual Review of Nutrition*.

[185] Mavroudi A., Xinias I. (2011). Dietary interventions for primary allergy prevention in infants. *Hippokratia Medical Journal*.

[186] Roberfroid M., Gibson G. R., Hoyles L. (2010). Prebiotic effects: metabolic and health benefits. *British Journal of Nutrition*.

[187] Tanaka M., Nakayama J. (2017). Development of the gut microbiota in infancy and its impact on health in later life. *Allergology International*.

[188] Bode L. (2015). The functional biology of human milk oligosaccharides. *Early Human Development*.

[189] German J. B., Freeman S. L., Lebrilla C. B., Mills D. A. (2008). Human milk oligosaccharides: evolution, structures and bioselectivity as substrates for intestinal bacteria. *Personalized Nutrition for the Diverse Needs of Infants and Children*.

[190] Austin S., De Castro C., Bénet T. (2016). Temporal change of the content of 10 oligosaccharides in the milk of Chinese urban mothers. *Nutrients*.

[191] Boehm G., Müller-Werner B., Jelinek J., Stahl B. (2010). Variation of human milk oligosaccharides in relation to milk groups and lactational periods. *British Journal of Nutrition*.

[192] Akkerman R., Faas M. M., de Vos P. (2019). Non-digestible carbohydrates in infant formula as substitution for human milk oligosaccharide functions: effects on microbiota and gut maturation. *Critical Reviews in Food Science and Nutrition*.

[193] Bakker-Zierikzee A. M., Alles M. S., Knol J., Kok F. J., Tolboom J. J. M., Bindels J. G. (2005). Effects of infant formula containing a mixture of galacto- and fructo-oligosaccharides or viable Bifidobacterium animalis on the intestinal microflora during the first 4 months of life. *British Journal of Nutrition*.

[194] Ackerman D. L., Craft K. M., Townsend S. D. (2017). Infant food applications of complex carbohydrates: structure, synthesis, and function. *Carbohydrate Research*.

[195] Macfarlane G. T., Steed H., Macfarlane S. (2008). Bacterial metabolism and health-related effects of galacto-oligosaccharides and other prebiotics. *Journal of Applied Microbiology*.

[196] Urashima T., Taufik E., Fukuda K., Asakuma S. (2013). Recent advances in studies on milk oligosaccharides of cows and other domestic farm animals. *Bioscience, Biotechnology, and Biochemistry*.

[197] Vandenplas Y., De Greef E., Veereman G. (2014). Prebiotics in infant formula. *Gut microbes*.

[198] Vandenplas Y., Zakharova I., Dmitrieva Y. (2015). Oligosaccharides in infant formula: more evidence to validate the role of prebiotics. *British Journal of Nutrition*.

[199] Hoffen E., van Ruiter B., Faber J. (2009). A specific mixture of short-chain galacto-oligosaccharides and long-chain fructo-oligosaccharides induces a beneficial immunoglobulin profile in infants at high risk for allergy. *Allergy*.

[200] Rijnierse A., Jeurink P. V., van Esch B. C. A. M., Garssen J., Knippels L. M. J. (2011). Food-derived oligosaccharides exhibit pharmaceutical properties. *European Journal of Pharmacology*.

[201] Singh R. S., Singh R. P., Kennedy J. F. (2016). Recent insights in enzymatic synthesis of fructooligosaccharides from inulin. *International Journal of Biological Macromolecules*.

[202] Zolnere K., Ciprovica I. (2017). The comparison of commercially available *β*-galactosid22ases for dairy industry: review. *Food Science*.

[203] Closa-Monasterolo R., Gispert-Llaurado M., Luque V. (2013). Safety and efficacy of inulin and oligofructose supplementation in infant formula: results from a randomized clinical trial. *Clinical Nutrition*.

[204] Sabater C., Prodanov M., Olano A., Corzo N., Montilla A. (2016). Quantification of prebiotics in commercial infant formulas. *Food Chemistry*.

[205] Hill C., Guarner F., Reid G. (2014). The International Scientific Association for Probiotics and Prebiotics consensus statement on the scope and appropriate use of the term probiotic. *Nature Reviews Gastroenterology & Hepatology*.

[206] Bergmann H., Rodríguez J. M., Salminen S., Szajewska H. (2014). Probiotics in human milk and probiotic supplementation in infant nutrition: a workshop report. *British Journal of Nutrition*.

[207] Slavin J. (2013). Fiber and prebiotics: mechanisms and health benefits. *Nutrients*.

[208] Nagpal R., Kumar A., Kumar M., Behare P. V., Jain S., Yadav H. (2012). Probiotics, their health benefits and applications for developing healthier foods: a review. *FEMS Microbiology Letters*.

[209] Davis E. C., Dinsmoor A. M., Wang M., Donovan S. M. (2020). Microbiome composition in pediatric populations from birth to adolescence: impact of diet and prebiotic and probiotic interventions. *Digestive Diseases and Sciences*.

[210] Boesten R., Schuren F., Amor K. B., Haarman M., Knol J., de Vos W. M. (2011). Bifidobacterium population analysis in the infant gut by direct mapping of genomic hybridization patterns: potential for monitoring temporal development and effects of dietary regimens. *Microbial Biotechnology*.

[211] Braegger C., Chmielewska A., Decsi T. (2011). Supplementation of infant formula with probiotics and/or prebiotics: a systematic review and comment by the ESPGHAN committee on nutrition. *Journal of Pediatric Gastroenterology and Nutrition*.

[212] Hascoët J. M., Hubert C., Rochat F. (2011). Effect of formula composition on the development of infant gut microbiota. *Journal of Pediatric Gastroenterology and Nutrition*.

[213] Barile D., Rastall R. A. (2013). Human milk and related oligosaccharides as prebiotics. *Current Opinion in Biotechnology*.

[214] Cilla A., Lacomba R., Garcia-Llatas G., Alegria A. (2012). Prebiotics and nucleotides in infant nutrition: review of the evidence. *Revista Nutrición Hospitalaria*.

[215] Szajewska H., Chmielewska A. (2013). Growth of infants fed formula supplemented with 4146 Bifidobacterium lactis Bb12 or Lactobacillus GG: a systematic review of randomized controlled trials. *BMC Pediatrics*.

[216] Strömqvist M., Falk P., Hansson S. B. L., Lönnerdal B., Normark S., Hernell O. (1995). Human milk K-casein and inhibition of Helicobacter pylori adhesion to human gastric mucosa. *Journal of Pediatric Gastroenterology and Nutrition*.

[217] Shu X. O., Linet M. S., Steinbuch M. (1999). Breast-feeding and risk of childhood acute leukemia. *JNCI Journal of the National Cancer Institute*.

[218] Mirmiran M., Van Someren E. (1993). Symposium: Normal and abnormal REM sleep regulation: the importance of REM sleep for brain maturation. *Journal of Sleep Research*.

[219] Pellegrini A., Thomas U., Bramaz N. (1999). Isolation and identification of three bactericidal domains in the bovine Î±-lactalbumin molecule. *Biochimica et Biophysica Acta (BBA) - General Subjects*.

[220] Brück W. M., Graverholt G., Gibson G. R. (2002). Use of batch culture and a two-stage continuous culture system to study the effect of supplemental Î±-lactalbumin and glycomacropeptide on mixed populations of human gut bacteria. *FEMS Microbiology Ecology*.

[221] Brück W. M., Graverholt G., Gibson G. R. (2003). A two-stage continuous culture system to study the effect of supplemental alpha-lactalbumin and glycomacropeptide on mixed cultures of human gut bacteria challenged with enteropathogenic Escherichia coli and Salmonella serotype Typhimurium. *Journal of Applied Microbiology*.

[222] Lönnerdal B., Erdmann P., Thakkar S. K., Sauser J., Destaillats F. (2017). Longitudinal evolution of true protein, amino acids and bioactive proteins in breast milk: a developmental perspective. *The Journal of Nutritional Biochemistry*.

[223] Gustafsson L., Hallgren O., Mossberg A. K. (2005). HAMLET kills tumor cells by apoptosis: structure, cellular mechanisms, and therapy. *The Journal of Nutrition*.

[224] Puthia M., Storm P., Nadeem A., Hsiung S., Svanborg C. (2014). Prevention and treatment of colon cancer by peroral administration of HAMLET (human *α*-lactalbumin made lethal to tumour cells). *Gut*.

[225] Noteborn M. H. (2009). Proteins selectively killing tumor cells. *European Journal of Pharmacology*.

[226] Jiang R., Lopez V., Kelleher S. L., Lönnerdal B. (2011). Apo- and holo-lactoferrin are both internalized by lactoferrin receptor via clathrin-mediated endocytosis but differentially affect ERK-signaling and cell proliferation in caco-2 cells. *Journal of Cellular Physiology*.

[227] González-Chávez S. A., Arévallo-Gallegos S., Rascón-Cruz Q. (2009). Lactoferrin: structure, function and applications. *International Journal of Antimicrobial Agents*.

[228] Hagiwara T., Shinoda I., Fukuwatari Y., Shimamura S. (1995). Effects of lactoferrin and its peptides on proliferation of rat intestinal epithelial cell line, IEC-18, in the presence of epidermal growth factor. *Bioscience, Biotechnology, and Biochemistry*.

[229] Buccigrossi V., de Marco G., Bruzzese E. (2007). Lactoferrin induces concentration-dependent functional modulation of intestinal proliferation and differentiation. *Pediatric Research*.

[230] Turroni F., Milani C., Duranti S. (2020). The infant gut microbiome as a microbial organ influencing host well-being. *Italian Journal of Pediatrics*.

[231] Liepke C., Adermann K., Raida M., Mägert H.-J., Forssmann W.-G., Zucht H.-D. (2002). Human milk provides peptides highly stimulating the growth of bifidobacteria. *European Journal of Biochemistry*.

[232] Chen Y., Zheng Z., Zhu X. (2015). Lactoferrin promotes early neurodevelopment and cognition in postnatal piglets by upregulating the BDNF signaling pathway and polysialylation. *Molecular Neurobiology*.

[233] Pribylova J., Krausova K., Kocourkova I. (2012). Colostrum of healthy mothers contains broad spectrum of secretory IgA autoantibodies. *Journal of Clinical Immunology*.

[234] Rogier E. W., Frantz A. L., Bruno M. E. C. (2014). Secretory antibodies in breast milk promote long-term intestinal homeostasis by regulating the gut microbiota and host gene expression. *Proceedings of the National Academy of Sciences*.

[235] Wu G. (2020). Important roles of dietary taurine, creatine, carnosine, anserine and 4-hydroxyproline in human nutrition and health. *Amino acids*.

[236] Tochitani S. (2017). Functions of maternally-derived taurine in fetal and neonatal brain development. *Advances in Experimental Medicine and Biology*.

[237] Sturman J. A. (1988). Taurine in Development. *The Journal of Nutrition*.

[238] Sturman J. A., Rassin D. K., Gaul G. E. (1977). Taurine in developing rat brain: transfer of [35s] taurine to pups via the milk. *Pediatric Research*.

[239] Sturman J. A., Gaull G. E. (1975). Taurine in the brain and liver of the developing human and monkey. *Journal of Neurochemistry*.

[240] Chesney R. W., Helms R. A., Christensen M., Budreau A. M., Han X., Sturman J. A. (1998). The role of taurine in infant nutrition. *Taurine*.

[241] Vandeqoude M. F., Delleeuw L. H. (1985). Taurine requirement with parenteral nutrition. *The New England Journal of Medicine*.

[242] Wharton B. A., Morley R., Isaacs E. B., Cole T. J., Lucas A. (2004). Low plasma taurine and later neurodevelopment. *Archives of Disease in Childhood*.

[243] Bailey L. B., Stover P. J., McNulty H. (2015). Biomarkers of nutrition for development-folate review. *The Journal of Nutrition*.

[244] Haiden N., Klebermass K., Cardona F. (2006). A randomized, controlled trial of the effects of adding vitamin B12 and folate to erythropoietin for the treatment of anemia of prematurity. *Pediatrics*.

[245] Tâmega I. E., Costa C. D. (2007). Serum folic status in eutrophic infants nourished with breast milk, cow milk or milk modified formula. *Revista Paulista de Pediatria*.

[246] Veena S. R., Krishnaveni G. V., Srinivasan K. (2010). Higher maternal plasma folate but not vitamin B-12 concentrations during pregnancy are associated with better cognitive function scores in 9- to 10- year-old children in South India. *The Journal of nutrition*.

[247] Strand T. A., Taneja S., Bhandari N. (2007). Folate, but not vitamin B-12 status, predicts respiratory morbidity in north Indian children. *The American Journal of Clinical Nutrition*.

[248] Hure A. J., Collins C. E., Smith R. (2012). A longitudinal study of maternal folate and vitamin b12 status in pregnancy and postpartum, with the same infant markers at 6 months of age. *Maternal and Child Health Journal*.

[249] Nygren-Babol L., Jägerstad M. (2012). Folate-binding protein in milk: a review of biochemistry, physiology, and analytical methods. *Critical Reviews in Food Science and Nutrition*.

[250] Hay G., Johnston C., Whitelaw A., Trygg K., Refsum H. (2008). Folate and cobalamin status in relation to breastfeeding and weaning in healthy infants. *The American Journal of Clinical Nutrition*.

[251] Donangelo C. M., Trugo N. M. F., Koury J. C. (1989). Iron, zinc, folate and vitamin B12 nutritional status and milk composition of low-income Brazilian mothers. *European Journal of Clinical Nutrition*.

[252] Foged N., Lillquist K., Rolschau J., Blaabjerg O. (1989). Effect of folic acid supplementation on small-for-gestational-age infants born at term. *European Journal of Pediatrics*.

[253] O’Connor D. L., Green T., Picciano M. F. (1997). Maternal folate status and lactation. *Journal of Mammary Gland Biology and Neoplasia*.

[254] Fang T., Liu G., Cao W. (2016). Spermine: new insights into the intestinal development and serum antioxidant status of suckling piglets. *RSC Advances*.

[255] Peulen O., Dewé W., Dandrifosse G., Henrotay I., Romain N. (1998). The relationship between spermine content of human milk during the first postnatal month and allergy in children. *Public Health Nutrition*.

[256] Bruun S., van Rossem L., Lauritzen L. (2019). Content of n-3 LC-PUFA in breast milk four months postpartum is associated with infancy blood pressure in boys and infancy blood lipid profile in girls. *Nutrients*.

[257] Liu J. J., Green P., John Mann J., Rapoport S. I., Sublette M. E. (2015). Pathways of polyunsaturated fatty acid utilization: implications for brain function in neuropsychiatric health and disease. *Brain Research*.

[258] Willatts P. (2018). Effects of nutrition on the development of higher-order cognition. *Nestle Nutrition Institute workshop series*.

[259] Innis S. M., Gilley J., Werker J. (2001). Are human milk long-chain polyunsaturated fatty acids related to visual and neural development in breast-fed term infants?. *The Journal of Pediatrics*.

[260] Jensen C. L., Voigt R. G., Prager T. C. (2005). Effects of maternal docosahexaenoic acid intake on visual function and neurodevelopment in breastfed term infants. *The American Journal of Clinical Nutrition*.

[261] O'Connor D. L., Hall R., Adamkin D. (2001). Growth and development in preterm infants fed long-chain polyunsaturated fatty acids: a prospective randomized controlled trial. *Pediatrics*.

[262] Agostoni C., Braegger C., Decsi T. (2008). Breastfeeding: a commentary by the ESPGHAN Committee on Nutrition. *Journal of Pediatric Gastroenterology and Nutrition*.

[263] Le Huërou-Luron I., Blat S., Boudry G. (2010). Breast- v. formula-feeding: impacts on the digestive tract and immediate and long-term health effects. *Nutrition Research Reviews*.

[264] Murphy K., Curley D., O'Callaghan T. F. (2017). The composition of human milk and infant faecal microbiota over the first three months of life: a pilot study. *Scientific Reports*.

[265] Cerdó T., Diéguez E., Campoy C. (2019). Infant growth, neurodevelopment and gut microbiota during infancy. *Current Opinion in Clinical Nutrition and Metabolic Care*.

[266] Hoddinott P., Tappin D., Wright C. (2008). Breast feeding. *BMJ*.

[267] Chichlowski M., De Lartigue G., German J. B., Raybould H. E., Mills D. A. (2012). Bifidobacteria isolated from infants and cultured on human milk oligosaccharides affect intestinal epithelial function. *Journal of Pediatric Gastroenterology and Nutrition*.

[268] Huynh H. Q., deBruyn J., Guan L. (2009). Probiotic preparation VSL#3 induces remission in children with mild to moderate acute ulcerative colitis: a pilot study. *Inflammatory Bowel Diseases*.

[269] Miele E., Pascarella F., Giannetti E., Quaglietta L., Baldassano R. N., Staiano A. (2009). Effect of a probiotic preparation (VSL#3) on induction and maintenance of remission in children with ulcerative colitis. *The American Journal of Gastroenterology*.

[270] Guandalini S., Magazzù G., Chiaro A. (2010). VSL#3 improves symptoms in children with irritable bowel syndrome: a multicenter, randomized, placebo-controlled, double-blind, crossover study. *Journal of Pediatric Gastroenterology and Nutrition*.

[271] Gareau M. G., Sherman P. M., Walker W. A. (2010). Probiotics and the gut microbiota in intestinal health and disease. *Nature reviews Gastroenterology & Hepatology*.

[272] Alfaleh K., Anabrees J., Bassler D. (2010). Probiotics reduce the risk of necrotizing enterocolitis in preterm infants: a meta-analysis. *Neonatology*.

[273] Deshpande G., Rao S., Patole S. (2010). Updated meta-analysis of probiotics for preventing necrotizing enterocolitis in preterm neonates. *Pediatrics*.

[274] Lin H.-C., Hsu C.-H., Chen H.-L. (2008). Oral probiotics prevent necrotizing enterocolitis in very low birth weight preterm infants: a multicenter, randomized, controlled trial. *Pediatrics*.

[275] Ashley C., Johnston W. H., Harris C. L., Stolz S. I., Wampler J. L., Berseth C. L. (2012). Growth and tolerance of infants fed formula supplemented with polydextrose (PDX) and/or galactooligosaccharides (GOS): double-blind, randomized, controlled trial. *Nutrition Journal*.

[276] Birch E. E., Garfield S., Hoffman D. R., Uauy R., Birch D. G. (2000). Um ensaio clínico randomizado e controlado de suprimento alimentar precoce de ácidos graxos poliinsaturados de cadeia longa e desenvolvimento mental em bebês nascidos a termo. *Developmental Medicine & Child Neurology*.

[277] Cao X., Kang S., Yang M. (2018). Quantitative N-glycoproteomics of milk fat globule membrane in human colostrum and mature milk reveals changes in protein glycosylation during lactation. *Food & Function*.

[278] Colombo J., Carlson S. E., Cheatham C. L., Fitzgerald-Gustafson K. M., Kepler A., Doty T. (2011). Long-chain polyunsaturated fatty acid supplementation in infancy reduces heart rate and positively affects distribution of attention. *Pediatric research*.

[279] Escribano J., Ferré N., Gispert-Llaurado M. (2018). Bifidobacterium longum subsp infantis CECT7210-supplemented formula reduces diarrhea in healthy infants: a randomized controlled trial. *Pediatric Research*.

[280] Foiles A. M., Kerling E. H., Wick J. A., Scalabrin D. M. F., Colombo J., Carlson S. E. (2016). Formula with long-chain polyunsaturated fatty acids reduces incidence of allergy in early childhood. *Pediatric Allergy and Immunology*.

[281] Le Huërou-Luron I., Bouzerzour K., Ferret-Bernard S. (2018). A mixture of milk and vegetable lipids in infant formula changes gut digestion, mucosal immunity and microbiota composition in neonatal piglets. *European Journal of Nutrition*.

[282] Miklavcic J. J., Larsen B. M., Mazurak V. C. (2017). Reduction of arachidonate is associated with increase in B-cell activation marker in infants: a randomized trial. *Journal of Pediatric Gastroenterology and Nutrition*.

[283] Moro G., Arslanoglu S., Stahl B., Jelinek J., Wahn U., Boehm G. (2006). A mixture of prebiotic oligosaccharides reduces the incidence of atopic dermatitis during the first six months of age. *Archives of Disease in Childhood*.

[284] Oropeza-Ceja L. G., Rosado J. L., Ronquillo D. (2018). Lower protein intake supports normal growth of full-term infants fed formula: a randomized controlled trial. *Nutrients*.

[285] Simeoni U., Berger B., Junick J. (2016). Gut microbiota analysis reveals a marked shift to bifidobacteria by a starter infant formula containing a synbiotic of bovine milk‐derived oligosaccharides and B ifidobacterium animalis subsp. lactis CNCM I ‐3446. *Environmental Microbiology*.

