# Supplementary material for: Bioactive Compounds in Infant Formula and Their Effects on Infant Nutrition and Health: A Systematic Literature Review
Source: Int J Food Sci. 2021 May 14;2021:8850080. doi: 10.1155/2021/8850080 (PMC8140835; doi:10.1155/2021/8850080)
Supplement: Supplementary Materials — can be found at (link to manuscript tracking). Table S1: biological functions of bioactive compounds and recommendations to marketed infant formulas (regulated and unregulated compounds). Table S2: main clinical findings related to the enrichment of infant formulas with bioactive compounds and their effects on infant health. [file 8850080.f1.zip › Table S1 - Supplementary file_Biological functions of bioactive compounds.docx]

**Table S1.** Biological functions of bioactive compounds and recommendations to marketed infant formulas (regulated and unregulated compounds).

| Bioactive compounds | Biological functions | Infant formula | References |
| --- | --- | --- | --- |
| *β‐Casein* | - Ca, Zn, and P absorption - Opioid activities | Not added No regulation | Enjapoori et al., 2019 Jarmołowska et al., 2007a Ledesma-Martínez et al., 2019 Lönnerdal, 2016 Meisel & Gerald, 2003 Miquel et al., 2006 Wada & Lönnerdal, 2014 |
| *κ‐Casein* | - Immunomodulatory activity - Antimicrobial activity - Prebiotic | Not added No regulation | Bruck et al., 2003 Hernández-Ledesma et al., 2007 Liao et al., 2012 Sánchez & Vázquez, 2017 |
| *α‐Casein* | - Opioid activities | Not added No regulation | Hebert et al., 2010 Ledesma-Martínez et al., 2019 Liao et al., 2012 Wada & Lönnerdal, 2014 |
| *α‐Lactalbumin* | - Zn and Fe absorption - Immunomodulation - Antimicrobial activity - Prebiotics | Not added No regulation | Brück et al., 2003 Fleddermann et al., 2013 Golinelli et al., 2014 Kamau et al., 2012 Layman et al., 2018 Layman et al., 2018 Lönnerdal & Lien, 2003 Sandström et al., 2008 Szymlek-Gay et al., 2012 Trabulsi et al., 2011 Wada & Lönnerdal, 2014 |
| *Lactoferrin* | - Fe absorption - Immunomodulation - Antimicrobial activity - Intestinal development - Antiviral activity - Antioxidant activity - Cognitive development - Prebiotics | Not added No regulation | Johnston et al., 2015 King et al., 2007 Lönnerdal et al., 2011 Lönnerdal, 2009 Lönnerdal, 2014 Lönnerdal, 2017 Manzoni et al., 2009 Queiroz et al., 2013 Telang, 2018  Ballard & Morrow, 2013  Donavan, 2019  Haschke et al., 2016 |
| *Lysozyme* | - Antimicrobial activity | Not added No regulation | Maga et al., 2012 Hendricks & Guo, 2014 Lönnerdal, 2014 Lönnerdal, 2016 Lönnerdal, 2017 |
| *Secretory IgA* | - Immunomodulation - Antimicrobial activity | Not added No regulation | Ballard & Morrow, 2013  Basha et al., 2014 Hendricks & Guo, 2014  Donavan, 2019  Haschke et al., 2016 |
| *Taurine* | - Neurodevelopment - Neuroprotection - Anti-oxidative activity | Optional addition (max. 12 mg/100 mL) | Bouckenooghe et al., 2006  Cao et al., 2018  Kilb & Fukuda, 2017 Ripps & Shen, 2012 Roysommuti & Wyss, 2014  Manzi & Pizzoferrato, 2012  Tochitani, 2017  Verner et al., 2007  Wu, 2020 |
| *Folates* | - Cell replication (formation and maturation of red blood cells) | Must contain (10 - 50 µg/100 mL) | Ami et al., 2016  Bailey et al., 2016  Black et al., 2008  Czeizel et al., 2013  Haiden et al., 2006  Hay et al., 2008  Hure et al., 2011  Lamers, 2011 Ohrvik & Witthoft, 2011  Nygren-Babol & Jägerstad, 2012 |
| *Polyamines* | - Maturation of (liver and pancreas) - Immunomodulation - Intestinal development - Protein synthesis | Not added No regulation | Atiya et al., 2014 Bjelakovic et al., 2012 Büyükuslu, 2015 Garwolińska et al., 2018 Gómez-Gallego et al., 2012 Gómez-Gallego et al., 2014 Gómez-Gallego et al., 2017 Gómez-Gallego et al., 2019 Larqué et al., 2007 Pérez-Cano et al., 2010 Plaza-Zamora et al., 2013 Sabater-Molina et al., 2009 |
| *MFGM* | - Immune health - Intestinal immune maturation - Neurodevelopment (brain development and cognitive function) - Antiviral - Antibacterial activities | Not added No regulation | Bourlieu et al., 2015 Cao et al., 2018 Gurnida et al., 2012 Hernell et al., 2016 Koletzko, 2016 Le Huërou-Luron et al., 2018 Lee et al., 2020 McJarrow et al., 2009  Nelly et al., 2011  Nieto-Ruiz et al., 2019  Ortega-Anaya & Jimenez-Flores, 2019 Ran-Ressler et al., 2011 Tanaka et al., 2013 Timby et al., 2014 Timby et al., 2015 |
| *LC-PUFA* | - Immunomodulation - Neurodevelopment | Lipid (3-6 g/100 Kcal).  Linoleic acid (300-1,200 mg/100 kcal), or a range of 7% - 20% of total fatty acids.  LC-PUFAs - Optional addition (max. level 2% of total fatty acids for ARA and 1% for DHA), except for European countries where the addition is mandatory (20–50 mg/100 mL) | Birch et al., 2007  Bruun et al., 2019  Calder, 2016  Colombo et al., 2011  Echeverría et al., 2017  Fleddermann et al., 2014  Foiles et al., 2016  Koletzko, 2016  Lien et al., 2018  Miklavcic et al., 2017  Nieto-Ruiz et al., 2019  Pastor et al., 2006  Rogers et al., 2014  Tinoco et al., 2007 Lapillonne et al., 2014 Echeverría et al., 2017 Qawasmi et al., 2012 Qawasmi et al., 2013 Thompkinson et al., 2007 |
| *Prebiotic* | - Modulate the intestinal microbiota composition - Prevent pathogen adhesion - Immunoreactivity modulation (preventing allergic responses or food hypersensitivity) | Optional nutrient (9:1 ratio of GOS:FOS - max. 0.8 g/dL ) | Ashley et al., 2012  Boehm & Stahl, 2007 Smilowitz et al., 2014 Elwakiel et al., 2018 Van den Broek et al., 2008 Roberfroid et al., 2010 Mavroudi & Xinias, 2011 Tanaka & Nakayama, 2017  Closa-Monasterolo et al., 2013  Huffen et al., 2009  [Moro](https://pubmed.ncbi.nlm.nih.gov/?term=Moro+G&cauthor_id=16873437) et al., 2006 |
| *Probiotic* | - Modulate the intestinal microbiota composition - Production of antimicrobial compounds - Bioavailability and digestibility of lipids and proteins - Ca, P, Fe absorption - B vitamins' synthesis | Optional nutrient. Viable bacteria (*Lactobacillus* and/or *Bifidobacterium*) should be between 106 to 108 CFU/g of the product ready for consumption. | Escribano et al., 2018  Hascoët et al., 2011  Li et al., 2019  Simeoni et al., 2016  Slavin, 2013 Bergmann et al., 2014 Nagpal et al., 2012 Barile & Rastall, 2013 Thomson et al., 2018 Slavin, 2013 |
| Ca, calcium; Zn, zinc; P, phosphor; MFGM, milk fat globule membrane; LC-PUFAs, Long-chain polyunsaturated fatty acids; DHA, docosahexanoic acid; ARA, arachidonic acid; GOS, galacto-oligosaccharides; FOS, fructo-oligosaccharides; Fe, iron. | | | |
