# Supplementary material for: Bioactive Compounds in Infant Formula and Their Effects on Infant Nutrition and Health: A Systematic Literature Review
Source: Int J Food Sci. 2021 May 14;2021:8850080. doi: 10.1155/2021/8850080 (PMC8140835; doi:10.1155/2021/8850080)
Supplement: Supplementary Materials — can be found at (link to manuscript tracking). Table S1: biological functions of bioactive compounds and recommendations to marketed infant formulas (regulated and unregulated compounds). Table S2: main clinical findings related to the enrichment of infant formulas with bioactive compounds and their effects on infant health. [file 8850080.f1.zip › Table S2 - Supplementary file_Main clinical findings.docx]

**Table S2.** Main clinical findings related to the enrichment of infant formulas with bioactive compounds and their effects on infant health.

| Reference | Bioactive Compounds | Background & aims | Age | Disease state | Sample size | Main findings |
| --- | --- | --- | --- | --- | --- | --- |
| Fleddermann et al., 2014 | α-Lactoalbumin  L-PUFA | Protein source and content of LC-PUFA of EF may influence infant growth. The aim was to assess the effect of a modified IF on growth. | Term infants | Healthy infants | 213 | There was no difference between weight gain between groups of formulas. However, the gain in length was higher in the IF, and growth per energy intake was higher in IF than SF for weight and length. |
| King et al. 2007 | Lactoferrin | Lactoferrin has bioactivity that includes growth, immune modulation, and antimicrobial effects. The aim was to assess the impact of long-term feeding using formulas enriched with lactoferrin on the growth, hematology, immune parameters, and reduction in the incidence of respiratory diseases. | 0-4 wk | NA | 52 | There is no significant difference in growth parameters, a trend toward increased weight gain, better hematological parameters, fewer lower respiratory tract illnesses than infants fed with SF. |
| Davis et al., 2008 | α-Lactoalbumin | Evaluated an α-La enriched formula's efficacy and safety with a protein profile and total protein concentration closer to HM and lower than SF. | Term infants | Healthy infants | 216 | The formula enriched with α-La delivered lower total protein with higher protein quality, as demonstrated by the plasma essential amino-acids profiles compared to the group that received SF. Furthermore, the α-La enriched formula demonstrated improved gastrointestinal tolerance over SF with significantly fewer gastrointestinal events and a similarity in the cumulative incidence and timing of gastrointestinal study events to breastfed infants. |
| Sandström et al., 2008 | κ-casein (GMP) α-Lactoalbumin | Adding α-La to IF has been proposed because it modifies the plasma amino acid pattern in infants, making it more similar to that of breastfed infants. The addition of α-La to IF containing different GMP concentrations was proposed to modify the infant's plasma amino acid pattern, allowing a reduction in the formula's protein content, which can affect growth. | 6 ± 2 wk | NA | 96 | Compared with SF-fed infants, infants formula-fed with a modified protein composition had growth patterns more similar to those of breastfed infants. All formula-fed groups had plasma amino acid concentrations similar to or higher than those of breastfed infants. Moreover, the plasma tryptophan concentrations in infants fed enriched in α-La enhanced formulas were more similar to those of breastfed infants than those of infants SF-fed, which suggests that the protein quality of the formula was enhanced. |
| Brück etal., 2003 | κ-casein (GMP) α-Lactoalbumin | The addition of GMP or α-La helps promote the growth of a host-friendly colonic microflora (e.g., bifidobacteria, lactobacilli) and explains why breastfed infants experience fewer and milder intestinal infections than those who are formula-fed. The effects of supplementation of formula with these two bioactive proteins were investigated in this study. | 0-5 Mo  (infant rhesus macaques) | NA | 20 | Breastfed infants and infants fed α-La supplemented formula had no diarrhea, while those infants fed GMP-supplemented formula had intermittent diarrhea. In infants SF-fed, the diarrhea was acute. |
| Trabulsi et al., 2011 | α-Lactoalbumin | The protein concentration of infant feeding has a role in weight gain during infancy, affecting later life weight. The objective of the study was to evaluate the effect of an α-La enriched formula with a lower protein concentration on infant growth. | Term infants | Healthy infants | 336 | α-La enriched formula containing 12.8 g/L protein was safe and supported age-appropriate growth, and no difference in the incidence of study events compared with the SF and HM groups. |
| Oropeza-Ceja et al., 2018 | α-Lactoalbumin | The excess of proteins induces accelerated growth patterns, leading to the development of chronic diseases later in life. This study aimed to evaluate the safety of an IF enriched with bovine α-La containing a total protein concentration close to that of HM (1.0 g ptn/dL) and determine its effectiveness in supporting healthy infant growth in the 1 to 4 month of age. | Term infants | Healthy infants | 308 | Low protein EF delayed weight gain, similar to exclusively breastfed infants. |
| Johnston et al., 2015 | Lactoferrin | Lf is a multifunctional iron-binding protein predominant in HM, shares a similar protein sequence, structure, and bioactivity with bovine Lf. The study evaluated growth and tolerance in infants who received formulas with bovine Lf with a prebiotic blend (PDX) and galactooligosaccharides (GOS) in concentrations similar to that of mature HM. | 12-16 day-old infants | NA | 480 | Infants who received EF with bLf with a mixture of PDX and GOS presented a stronger bifidogenic effect and a smoother evacuation pattern, similar to that reported in breastfed infants. Furthermore, the study demonstrated that the EF was safe and well-tolerated and was associated with normal growth when administered to healthy term infants. |
| Manzoni et al., 2009 | Lactoferrin | bLf activity is enhanced by the probiotic *Lactobacillus* *rhamnosus* GG (LGG). This study aimed to establish whether bLf, alone or in combination with LGG, reduces late-onset sepsis incidence in very low birth weight neonates. | Premature neonates | Late-onset sepsis | 472 | Supplementation with bLf alone or in combination with LGG reduced the incidence of a first episode of late-onset sepsis in very low birth weight infants compared to placebo. |
| Troesch et al., 2019 | Folic acid 5-methyl-THF | 5-methyl-THF is the predominant form of folate in HM, but it is currently not approved as a folate source for IFs. The study aimed to assess the suitability of 5-methyl-THF as a folate source for children, evaluating their effect on growth, tolerability, and safety indicators in infants. | Term infants | Healthy infants | 360 | Infants who consumed an IF with 5-MTHF showed no significant growth and tolerance differences than infants fed the same formula with folic acid at equimolar doses. |
| Gómez-Gallego et al., 2012 | Polyamines | Polyamines play an essential role in developing the intestinal and immune systems in the early stage of breastfeeding. The study assessed the effect of IF supplementation with a mixture of polyamines (putrescine, spermidine and sperm) on the intestinal microbiota composition in neonatal BALB / coloHsd mice. | ~8 wk (mice) | Healthy mice | 48 | The results showed that polyamines in FI interacted with the microbiota development, and the microbiota composition in the groups with supplemented formula and were similar to the breastfeeding group. The most predominant groups found in mouse large intestinal contents are similar to those found in human infants. |
| Sabater-Molina et al., 2009 | Polyamines | Polyamines are essential for many cellular functions and are part of the composition of HM. The study aimed to evaluate the effects of IFs designed to resemble sow milk supplemented with polyamines on piglets' intestinal maturation weaned early. | Newborn piglets | NA | 30 | The results demonstrate that polyamine ingestion at physiologic doses by early-weaned piglets significantly affected the morphology of the small intestine but not all biochemical parameters associated with it, suggesting that dietary polyamines might be regarded as beneficial compounds for small intestinal growth and development. |
| Pérez-Cano et al., 2010 | Polyamines | The study evaluated whether supplementation with polyamines present in HM (spermine and spermidine) influenced the postnatal maturation of rats' systemic and intestinal immune system. | Newborn mice | NA | NA | The daily supplementation with either spermine or spermidine from the day of birth and continuously during suckling was able to enhance intestinal and systemic immune cells' maturation. Furthermore, it was concluded that polyamines' systemic effect increases innate immunity rather than acquired immune responses. |
| Gómez-Gallego et al., 2014 | Polyamines | It was evaluated whether the proportion of polyamine found in HM, administered with commercial IF, affected the maturation of the immune system in a BALB / coloHsd mouse model. | 2 wk  (mice) | NA | 48 | The study demonstrates that the enrichment of IF with polyamines enhanced the maturation's systemic and intestinal immune system. |
| Timby et al., 2017 | MFGM | Several individual components of MFGM have been shown to be essential for brain development. In this study, the health effects of infants fed an EF with reduced energy and protein content combined with supplementation with a bovine MFGM fraction were evaluated. | < 2 MO | Healthy infants | 160 | Infants fed with MFGM-supplemented EF performed better on cognitive testing at 12 mo than did infants fed SF and were similar to exclusively breastfed babies. Furthermore, EF compensated for reduced energy and protein densities and showed a growth pattern similar to babies fed with FS. |
| Timby et al., 2015 | MFGM | Formula-fed infants have a higher incidence of acute otitis media (AOM) and gastrointestinal and respiratory tract infections during the first year of life compared to breastfed infants. The study assessed whether that supplementing IFs with bovine MFGM decreases infectious morbidity in formula-fed infants. | < 2 MO | Healthy infants | 160 | During the intervention, the incidence of AOM was lower in the EF group than in the SF group and did not differ from the breastfed reference group. Also, it reduced the number of days of antipyretics, which corroborated the hypothesis that IFs supplemented with MFGM have a preventive effect on infections. |
| Gurnida et al., 2012 | MFGM (Gangliosides) | HM contains gangliosides which may play an important role in infant neurodevelopment. This study evaluated the impact of IF supplemented with gangliosides (components of MFGM) on the cognitive functions of normal infant healthy. | Term infants | Healthy infants | 110 | The supplementation of IF with gangliosides increased its serum levels, correlating with higher scores for hand and eye coordination, IQ of performance and General IQ, thus demonstrating positive effects on cognitive development. |
| Nelly et al., 2011 | MFGM | Proteins present in bovine MFGM have been shown to have broad activity against pathogens. This study evaluated the effectiveness of a formulation using a whey protein concentrate enriched with MFGM in diarrhea, anemia, and micronutrient status. | 6-11 MO | Healthy infants | 550 | The results show that the addition of a whey protein concentrate enriched with MFGM provided to infants reduced the probability of an episode of diarrhea with blood and the prevalence of diarrhea. |
| Li et al., 2019 | Probiotic MFGM | The consumption of IF containing F19 or MFGM seems to reduce the incidence of infections. This study evaluated the safety and effects on infections and growth of two IFs, one supplemented with the probiotic bacteria L. *paracasei,* ssp. *paracasei* (strain F19) and the other with the bovine MFGM fraction compared to an SF and an exclusively breastfed group. | Term infants | Healthy infants | 789 | Both formulas supplemented with MFGM and F19 were safe for weight gain and did not differ from the babies who received SF and the breastfed reference group. In general, the formulas were well-tolerated but did not show positive effects on the studied health outcomes. However, the MFGM group results were close to those of the breastfed group, supporting previous findings that show that supplementation of IFs with MFGM reduces the gap between breastfed and formula-fed infants concerning infections. |
| Le Huërou-Luron et al., 2018 | MFGM | The study evaluated the effect of adding MFGM to IFs on intestinal digestion, mucosal immunity and microbiota composition. | Newborn piglets | NA | 42 | The incorporation of MFGM in EF has been shown to modify protein digestion, the maturation dynamics of the immune system, and the fecal microbiota composition. |
| Lapillonne et al., 2014 | LC-PUFAs | LC-PUFAs can influence the immune system. This study aimed to compare the frequency of common diseases in infants who received formula with or without added LC-PUFAs. | Term infants | Healthy infants | 325 | Infants fed IF with DHA and ARA had a lower incidence and late-onset of respiratory diseases and symptoms of respiratory diseases and diarrhea requiring medical attention when compared to babies who received formula without DHA and ARA. |
| Nieto-Ruiz et al., 2019 | MFGM LC-PUFAs  (ARA and DHA)  Symbiotics  (FOS, inulin, *Bifidobacterium* and *L. rhamnosus*) | The influence of a new IF enriched with bioactive compounds (MFGM, symbiotics (FOS, inulin, *Bifidobacterium* and *Lactobacillus rhamnosus*), LC-PUFAs (ARA and DHA)) on growth, neurodevelopment and visual function was evaluated. | 0-18 MO | Healthy infants | 170 | The results suggested no significant growth differences between the study groups during the first 18 months of life. The visual function was improved in EF-fed infants compared with those that receive SF, like breastfed babies. |
| Pastor et al., 2006 | LC-PUFAs  (DHA and ARA) | LC-PUFAs may inhibit the allergic immune response through some anti-inflammatory properties. These studies determined whether supplementing IFs with DHA and ARA would reduce the incidence of respiratory symptoms (rhinitis and bronchiolitis) in the first year of life. | Term infants | Healthy infants | 1342 | The findings suggested that DHA / ARA supplementation may positively impact reducing diseases of the respiratory tract. The growth rates between the groups were similar; however, the treatment group had a lower incidence of bronchiolitis/bronchitis than the control group, and a lower incidence of rhinitis and a lower incidence of upper airway infection in the experimental group compared to the control group. |
| Foiles et al., 2016 | LC-PUFAs  (DHA and ARA) | LC-PUFAs can protect the immune system against the development of allergies. In this study, children who consumed EF supplemented with PUFA and children who consumed formulas without PUFA (control) were followed up. | Term infants | Healthy infants | 91 | Groups that consumed EF had a lower incidence of allergic diseases and skin allergy in the first year of life and longer time until the first allergic illness and possible reduced risks of allergic disease, wheezing, asthma in the experimental group. |
| Miklavcic et al., 2017 | LC-PUFAs  (ARA) | ARA status is associated with a decrease in immune function and is associated with a decrease in the B cell activation marker. The study aimed to determine whether the intake of ARA alters the levels of ARA of lymphocytes, plasma and red blood cells in babies fed IF. | Term infants | Healthy infants | 89 | Intake of ARA was not associated with the level of ARA in the lymphocytes in a dose-response manner, but ARA exerted an immunoregulatory role, decreasing the B cell activation markers and subsequent B cell functions in the immune system. Moreover, higher plasma levels of ARA were associated with increasing levels of ARA intake. |
| Birch et al., 2007 | LC-PUFAs  (DHA and ARA) | This study aimed to evaluate the supplementation of IFs with DHA and ARA in visual and cognitive results at four years of age. | Term infants | NA | 79 | At four years, the control formula group had poorer visual acuity than the breastfed group; the DHA- and DHA + ARA-supplemented groups did not differ significantly from the breastfed group. The control formula and DHA-supplemented groups had Verbal IQ scores poorer than the breastfed group. |
| Colombo et al., 2011 | LC-PUFAs  (DHA and ARA) | The level of DHA may affect learning and cognition. This study determined whether there was a dose-response for different supplementation levels of DHA and ARA in infants' cognitive performance (children's learning and attention). Heart rate was also assessed. | Term infants | Healthy infants | 122 | Children who were supplemented with the lowest doses of DHA spent more time processing the active stimulus (attention and learning) than babies fed the non-supplemented formula, while babies fed the highest dose were intermediate and did not differ from any other group. Children supplemented with DHA and ARA had lower heart rates than those not supplemented, with no dose-response for this effect. |
| Huffen et al., 2009 | GOS  FOS | HMOs play a role in developing the immune system in infants and can, consequently, inhibit the onset of allergies. The study was aimed to analyze the effect of GOS/FOS on the immune response in infants. | Term infants | NA | 84 | The study showed that GOS/FOS supplementation induced a beneficial antibody profile. Furthermore, GOS/FOS supplementation specifically modulated the immune response towards cows' milk allergens. |
| Closa-Monasterolo et al., 2013 | Prebiotic - Fructan formulation composed of approximately 50% oligofructose and 50% inulin. Orafti®Synergy1 (SYN1) | The type of diet can influence the colonization of the gastrointestinal tract. FIs try to mimic the bifidogenic effect of HM using supplementation with prebiotics. The study evaluated the efficacy, safety and tolerance of an EF supplemented with inulin enriched with oligofructose during the first four months of life. | Term infants | Healthy infants | 300 | This study demonstrated that SYN1 supplementation promoted a trend toward increased Bifidobacterium in the gut, in addition to being safe, effective and well tolerated during the first four months. |
| Hascoët et al., 2011 | Probiotic – *B. longum* (BL999) | Breast-feeding induces a gut microbiota rich in bifidobacteria, whereas formula-fed babies have more diverse colonization.  The study evaluated the bifidogenic effect of an EF with a low content of phosphate and protein. | Term infants | Healthy infants | 190 | Bifidobacteria counts were significantly higher in babies who received EF than the group who consumed SF and were similar to breastfed infants. However, there were no significant differences in measures of growth, digestive tolerance and adverse events between groups. |
| Ashley et al., 2012 | HMOs GOS | HMOs modulate the infant immune system as well as influence the development of the intestinal microbiota. The study evaluated the effect of FIs supplemented with a prebiotic mixture of PDX and GOS or GOS alone on babies' overall growth and tolerance between 14 to 120 days of age. | 12-16 day-old infants | Healthy infants | 419 | IFs supplemented with a prebiotic mixture of PDX and GOS or GOS alone were well tolerated and support normal growth. Compared with infants who received an unsupplemented control formula, infants who received prebiotic supplementation experienced a standard evacuation smoother than that reported in breastfed infants. |
| [Moro](https://pubmed.ncbi.nlm.nih.gov/?term=Moro+G&cauthor_id=16873437) et al., 2006 | FOS  GOS | Oligosaccharides may alter postnatal immune development by influencing the constitution of gastrointestinal bacterial flora. This study investigated the effect of a prebiotic mixture (GOS and FOS) on the incidence of atopic dermatitis during the first six months of life in IF-fed infants at high risk of atopy. | Term infants | High-risk infants for atopic dermatitis | 206 | Prebiotic supplements were associated with a significantly higher number of fecal *bifidobacteria* than controls, but there was no significant difference in the lactobacillus count. |
| Escribano et al., 2018 | Probiotic  (*B. infantis* IM1) | Bovine milk contains oligosaccharides, some of which are structurally identical or similar to those found in HM. The feeding effect was tested with a formula supplemented with a mixture of oligosaccharides derived from bovine milk generated from whey permeate, containing galactooligosaccharides and 3'- and 6'-sialylactose, and the probiotic *Bifidobacterium* *animalis* subsp. lactis strain CNCM I - 3446. | Term infants | Healthy infants | 115 | The addition of BMOs to FIs showed a stimulating effect of bifidobacteria as high or even greater than FOS/GOS. The EF was faster and more efficient in bringing the infant microbiota to a community dominated by bifidobacteria, even compared to the breastfed group. |
| Simeoni et al., 2016 | BMOS *B. lactis*  (CNCM I ‐ 3446) | The microbiota can play an important role in modulating health and disease throughout life. The intestinal microbiota of breastfed infants is plenty of beneficial bifidobacteria. | Term infants | Healthy infants | 190 | The formula supplemented with *B. infantil* has been shown to prevent episodes of diarrhea and has been associated with a lower incidence of constipation, as well as a higher frequency of stools compared to the control group. |
| IF, infant formula; HM, human milk; EF, experimental formula; SF, standard formula; Wks, weeks; Mo, months of age; GMP, Glycomacropeptide; bLf, bovine lactoferrin; hLf, human lactoferrin; MFGM, milk fat globule membrane; LC-PUFAs, Long-chain polyunsaturated fatty acids; DHA, docosahexanoic acid; ARA, arachidonic acid; HMOs, human milk oligosaccharides; GOS, galacto-oligosaccharides; FOS, fructo-oligosaccharides; NA, not applicable. | | | | | | |
